# Altered machinery of protein synthesis is region- and stage-dependent and is associated with α-synuclein oligomers in Parkinson’s disease

**DOI:** 10.1186/s40478-015-0257-4

**Published:** 2015-12-01

**Authors:** Paula Garcia-Esparcia, Karina Hernández-Ortega, Anusha Koneti, Laura Gil, Raul Delgado-Morales, Ester Castaño, Margarita Carmona, Isidre Ferrer

**Affiliations:** Institute of Neuropathology, Bellvitge University Hospital, University of Barcelona, Bellvitge Biomedical Research Institute (IDIBELL), Hospitalet de Llobregat; Biomedical Research Center of Neurodegenerative Diseases (CIBERNED), Barcelona, Spain; Department of Genetics, Medical School, Alfonso X el Sabio University, Villanueva de la Cañada, Madrid, Spain; Cancer Epigenetics and Biology Program, IDIBELL, Hospitalet de Llobregat, Barcelona, Spain; Biology-Bellvitge Unit, Scientific and Technological Centers-University of Barcelona (CCiTUB), Hospitalet de Llobregat, Barcelona, Spain; Institute of Neuropathology, Service of Pathologic Anatomy, Bellvitge University Hospital, carrer Feixa Llarga s/n, 08907, Hospitalet de Llobregat, Spain

**Keywords:** α-synuclein oligomers, Parkinson’s disease, protein synthesis, nucleolar stress, ribosomes

## Abstract

**Introduction:**

Parkinson’s disease (PD) is characterized by the accumulation of abnormal α-synuclein in selected regions of the brain following a gradient of severity with disease progression. Whether this is accompanied by globally altered protein synthesis is poorly documented. The present study was carried out in PD stages 1-6 of Braak and middle-aged (MA) individuals without alterations in brain in the substantia nigra, frontal cortex area 8, angular gyrus, precuneus and putamen.

**Results:**

Reduced mRNA expression of nucleolar proteins nucleolin (NCL), nucleophosmin (NPM1), nucleoplasmin 3 (NPM3) and upstream binding transcription factor (UBF), decreased NPM1 but not NPM3 nucleolar protein immunostaining in remaining neurons; diminished 18S rRNA, 28S rRNA; reduced expression of several mRNAs encoding ribosomal protein (RP) subunits; and altered protein levels of initiation factor eIF3 and elongation factor eEF2 of protein synthesis was found in the substantia nigra in PD along with disease progression. Although many of these changes can be related to neuron loss in the substantia nigra, selective alteration of certain factors indicates variable degree of vulnerability of mRNAs, rRNAs and proteins in degenerating sustantia nigra. NPM1 mRNA and 18S rRNA was increased in the frontal cortex area 8 at stage 5-6; modifications were less marked and region-dependent in the angular gyrus and precuneus. Several RPs were abnormally regulated in the frontal cortex area 8 and precuneus, but only one RP in the angular gyrus, in PD. Altered levels of eIF3 and eIF1, and decrease eEF1A and eEF2 protein levels were observed in the frontal cortex in PD. No modifications were found in the putamen at any time of the study except transient modifications in 28S rRNA and only one RP mRNA at stages 5-6. These observations further indicate marked region-dependent and stage-dependent alterations in the cerebral cortex in PD. Altered solubility and α-synuclein oligomer formation, assessed in total homogenate fractions blotted with anti-α-synuclein oligomer-specific antibody, was demonstrated in the substantia nigra and frontal cortex, but not in the putamen, in PD. Dramatic increase in α-synuclein oligomers was also seen in fluorescent-activated cell sorter (FACS)-isolated nuclei in the frontal cortex in PD.

**Conclusions:**

Altered machinery of protein synthesis is altered in the substantia nigra and cerebral cortex in PD being the frontal cortex area 8 more affected than the angular gyrus and precuneus; in contrast, pathways of protein synthesis are apparently preserved in the putamen. This is associated with the presence of α-synuclein oligomeric species in total homogenates; substantia nigra and frontal cortex are enriched, albeit with different band patterns, in α-synuclein oligomeric species, whereas α-synuclein oligomers are not detected in the putamen.

**Electronic supplementary material:**

The online version of this article (doi:10.1186/s40478-015-0257-4) contains supplementary material, which is available to authorized users.

## Introduction

Neurodegenerative diseases with abnormal protein aggregates are characterized by post-translational modifications of constitutive proteins which result in abnormal conformation, truncation, and eventual formation of fibrils that impair endoplasmic reticulum function and alter the ubiquitin-proteasome system and autophagy pathways, thereby leading to their accumulation in neurons and, in some conditions, in glial cells. Alzheimer’s disease (AD), Parkinson’s disease (PD), tauopathies, amyotrophic lateral sclerosis, and Huntington’s disease are among this extensive group of disorders in which specific intra- and extracellular protein aggregates, together with the production and accumulation of abnormal oligomeric species, lead to neurodegeneration and neuronal death. In spite of the advances in understanding of specific altered proteins causative of particular diseases, little attention has been paid to the process of total protein synthesis in these disorders. This information is nevertheless crucial, as possible alterations in protein synthesis may jeopardize multiple cellular functions, fuel neurodegeneration and neuron atrophy (i.e. loss of dendrites, synapses and axons), and lead eventually to cell death.

Several studies have demonstrated ribosomal dysfunction and impaired protein synthesis in AD [1–5]. However, little information is available about alterations in protein synthesis in PD. Abnormal morphology and disruption of the nucleolus and reduced nucleolin expression have been reported in the substantia nigra in PD [6–8] and related experimental models [8, 9]. Mutations in DJ1 causative of familial PD alter rRNA biogenesis [10]. Added to this limited input is the fact that nothing is known about the possible link between the machinery of protein synthesis and α-synuclein aggregates, particularly α-synuclein oligomers in PD.

For these reasons, the present study was designed to identify possible alterations in concatenated pathways commanding protein synthesis from the nucleolus to the ribosome in regions with variable vulnerability to PD, including the substantia nigra, frontal cortex area 8, angular gyrus, precuneus, and putamen, at different stages of disease progression. The study includes analysis of selected nucleolar proteins involved in rRNA synthesis, rRNA 18S and rRNA 28S, and mRNAs of ribosomal proteins. This is followed by analysis of protein expression of initiation translation and elongation factors of protein synthesis at the ribosome. Finally, whether alterations are associated with α-synuclein oligomers was assessed in total homogenate fractions and in FACS-isolated nuclei analysed with anti-α-synuclein oligomer-specific antibodies. Post-mortem human brain is not suitable for direct studies of protein synthesis using *in vitro* incorporation of labelled amino acids in proteins because of unpredictable individual variations probably related to pre-mortem status and post-mortem delay in tissue processing. For this reason, the present study does not explore protein synthesis in human PD samples but rather focuses directly on the vulnerability of molecules and pathways involved in protein synthesis in several brain regions at different stages of disease progression in human PD.

## Material and methods

### Human cases

Brain tissue was obtained from the Institute of Neuropathology HUB-ICO-IDIBELL Biobank and the Hospital Clinic-IDIBAPS Biobank following the guidelines of the Spanish legislation on this matter and the approval of the local ethics committees. The post-mortem interval between death and tissue processing was between 3 and 20 h. Pathological cases were categorized as having PD pathology (Lewy body disease pathology) stages 1 to 6 according to the nomenclature of Braak et al. [11]. Only typical cases according to the Braak classification were included. Cases with concomitant tauopathies, excepting Alzheimer’s disease-related pathology stages I-II/0-B [12], vascular disease, and metabolic syndrome were excluded from the present study. Middle-aged (MA) cases had not suffered from neurologic, psychiatric, or metabolic diseases (including metabolic syndrome), and did not have abnormalities in the neuropathological examination excepting sporadic Alzheimer’s disease-related pathology stages I-II/0-B of Braak and Braak.

In total, 122 brains including 44 MA and 78 cases with PD-related pathology were included in the present study. Incidental PD (iPD or incidental Lewy Body Disease iLBD) occurred in 13 cases (mostly stages 1, 2, and 3 of Braak). Pre-parkinsonian symptoms in iPD cases were not recorded. Regarding PD cases, all of them had been treated for their motor symptoms. The disease duration ranged from 6 to 16 years. The most common causes of death in the MA and PD cases were infections, neoplasia, and acute cardiac disease.

Five regions were examined for mRNA expression: frontal cortex area 8, substantia nigra, angular gyrus, precuneus, and putamen; the selection of these areas was based on their differing vulnerability to PD and to their accumulative involvement with disease progression. Number of cases, mean ages, and standard deviation for each group are summarized in Table 1. A summary of individual cases and methods used for all cases examined is shown in Additional file 1: Table S1. Most cases here analysed were also the subject of other studies [13–15].

**Table 1:** Summary of the number of cases, mean ages, and standard deviation (SD) of each group of samples used in the present study including substantia nigra, frontal cortex area 8, angular gyrus, precuneus, and putamen. MA: middle-aged; PD: Parkinson’s disease

	substantia nigra	frontal cortex	angular gyrus	precuneus	putamen
	Number (N)	Number (N)	Number (N)	Number (N)	Number (N)
	mean age ± SD	mean age ± SD	mean age ± SD	mean age ± SD	mean age ± SD
MA	N = 11	N = 16	N = 10	N = 11	N = 15
	65.67 ± 12.76	63.88 ± 12.65	60.90 ± 10.28	64.64 ± 15.09	70.27 ± 8.89
PD stages 1-2	N = 6	N = 2	-	-	-
	80.17 ± 9.20	73.50 ± 2.12			
PD stages 3-4	N = 22	N = 17	N = 8	N = 10	N = 7
	76.55 ± 6.93	70.12 ± 8.40	66.50 ± 6.52	76.80 ± 7.73	76.00 ± 13.10
PD stages 5-6	N = 17	N = 12	N = 4	N = 4	N = 2
	78.82 ± 6.15	77.83 ± 4.51	79.25 ± 3.40	81.25 ± 3.86	78.00 ± 1.41

### RNA purification

Purification of RNA from the substantia nigra, right frontal cortex area 8, angular gyrus, precuneus, and putamen was carried out using RNeasy Lipid Tissue Mini Kit (Qiagen, Hilden, Germany) following the protocol provided by the manufacturer and performing the optional DNase I digest to avoid extraction and later amplification of genomic DNA. The concentration of each sample was obtained from A260 measurements with a NanoDrop 2000 spectrophotometer (Thermo Scientific, Waltham, MA, USA), and RNA integrity was tested using the Agilent 2100 BioAnalyzer (Agilent, Santa Clara, CA, USA).

### Retrotranscription reaction

Retrotranscription reaction of RNA samples was carried out with the High-Capacity cDNA Archive kit (Applied Biosystems, Foster City, CA, USA) following the guidelines provided by the manufacturer, and using a Gene Amp® 9700 PCR System thermocycler (Applied Biosystems). A parallel reaction for one RNA sample was processed in the absence of reverse transcriptase to rule out DNA contamination.

### Real Time PCR

RT-qPCR was conducted in duplicate on cDNA samples obtained from the retrotranscription reaction using 1,000ηg of RNA, diluted 1:20 in 384-well optical plates (Kisker Biotech, Steinfurt, Germany) utilizing the ABI Prism 7900 HT Sequence Detection System (Applied Biosystems). Parallel amplification reactions were carried out using 20x TaqMan Gene Expression Assays and 2x TaqMan Universal PCR Master Mix (Applied Biosystems). TaqMan probes used in the study are shown in Additional file 2: Table S2. The reactions were performed using the following parameters: 50 °C for 2 min, 95 °C for 10 min, 40 cycles at 95 °C for 15 s, and 60 °C for 1 min. TaqMan PCR data were captured using the Sequence Detection Software (SDS version 2.2, Applied Biosystems). Subsequently, threshold cycle (CT) data for each sample were analysed with the double delta CT (ΔΔCT) method. First, delta CT (ΔCT) values were calculated as the normalized CT values for each target gene in relation to the endogenous controls β-glucuronidase (GUS-β) and X-prolylaminopeptidase (aminopeptidase P) 1 (XPNPEP1) for normalization [16, 17]. Second, ΔΔCT values were obtained with the ΔCT of each sample minus the mean ΔCT of the population of MA samples (calibrator samples). The fold-change was determined using the equation 2^-ΔΔCT^. These housekeeping genes were selected because they show no modifications in several neurodegenerative diseases in human post-mortem brain tissue [16, 17]. Similar results were obtained using GUS-β and XPNPEP1 as correctors; GUS-β was selected for representation.

### Statistical analysis

The normality of distribution of the mean fold-change values obtained by RT-qPCR for each region and stage between MA and PD cases were analysed with the Kolmogorov-Smirnov test. The non-parametric Mann–Whitney test was performed to compare each group when the samples did not follow a normal distribution, while the unpaired *t* test was used for normal variables. *t* test was used instead of one-way ANOVA when analyzing MA and PD cases in parallel in the same optical plate. Statistical analysis was performed with GraphPad Prism version 5.01 (La Jolla, CA, USA) and Statgraphics Statistical Analysis and Data Visualization Software version 5.1 (Warrenton, VA, USA). Differences between groups were considered statistically significant at *P*-values: *P < 0.05, **P < 0.01 and ***P < 0.001.

### Gel electrophoresis and western blotting

Samples of the substantia nigra including 14 MA and 14 PD cases of frontal cortex area 8 (0.1 g of tissue) were homogenized with a glass homogenizer in Mila lysis buffer (0.5 M Tris at pH 7.4 containing 0.5 methylenediaminetetracetic acid at pH 8.0, 5 M NaCl, 0.5 % Na doxicholic, 0.5 % Nonidet P-40, 1 mM phenylmethylsulfonyl fluoride, bi-distilled water) with protease and phosphatase inhibitor cocktails (Roche Molecular Systems, Pleasanton, CA, USA), and then centrifuged at 4 °C for 15 min at 13,000 rpm (ultracentrifuge Beckman with 70Ti rotor, CA, USA). Protein concentration was measured by Smartspect™plus spectrophotometer (Bio-Rad, CA, USA) using the Bradford method (Merck, Darmstadt, Germany). Samples containing 20 μg of protein and the standard Precision Plus Protein™ Dual Color (Bio-Rad) were loaded onto 10 % and 12 % acrylamide gels. Proteins were separated in sodium dodecylsulfate-polyacrylamide gel electrophoresis (SDS-PAGE) and electrophoretically transferred to nitrocellulose membranes using the Trans-Blot®Turbo™ transfer system (Bio-Rad) at 200 mA/membrane for 20 min. Non-specific bindings were blocked by incubation in 5 % albumin in Tris-buffered saline (TBS) containing 0.2 % Tween for 1 h at room temperature. After washing, the membranes were incubated at 4 °C overnight with several antibodies in TBS containing 5 % albumin and 0.2 % Tween. A list of the antibodies used is shown in Additional file 3: Table S3. Monoclonal antibody anti-β-actin diluted 1:30,000 (β-Actin, A5316; Sigma-Aldrich, St. Louis, MO, USA) was blotted for the control of protein loading. Afterwards, the membranes were incubated for 1 h with the appropriate HRP-conjugated secondary antibody (1:1,000, Dako, Glostrup, Denmark), and the immune complexes were visualized with a chemiluminescence reagent (ECL, Amersham, GE Healthcare, Buckinghamshire, UK). Densitometry of western blot bands was assessed with the TotalLab program (TotalLab Quant, Newcastle, UK) and subsequently analysed with GraphPad Prism by one-way ANOVA with *post hoc* Tukey’s student range test for multiple comparisons. We used one-way ANOVA instead of *t* test because each gel contained MA and different stages of PD cases. Differences were considered statistically significant with *P*-values: *P <0.05; **P <0.01; ***P <0.001.

### Immunohistochemistry, double-labelling immunofluorescence, and confocal microscopy

Immunohistochemical study of selected nucleolar proteins was performed on 4 μm-thick dewaxed paraffin sections of the substantia nigra. PD cases were analysed including 2 stage 1 cases (1 male and 1 female), 3 stage 3 males, 2 stage 4 males, and 3 stage 5 males. Tissue sections were boiled in citrate buffer for 20 min to retrieve antigenicity. Endogenous peroxidases were blocked with peroxidase (Dako, Glostrup, Denmark) followed by 10 % normal goat serum. The primary antibodies were mouse monoclonal anti-nucleophosmin (NPM1) and rabbit polyclonal anti-nucleoplasmin-3 (NPM3). A few sections of the substantia nigra and frontal cortex area 8 were incubated with anti-α-synuclein oligomer-specific antibody (Agrisera, Vännäs, Sweden) at a dilution of 1:1,000. Following incubation with the primary antibody at room temperature overnight, the sections were incubated with EnVision + system peroxidase (Dako) at room temperature for 15 min. The peroxidise reaction was visualized with diaminobenzidine (DAB) and H_2_O_2_. The omission of the primary antibody in some sections was used as a control for the immunostaining; no signal was obtained with the incubation only of the secondary antibody. No immunogenic peptides were available for any antibody used. Sections were slightly counterstained with haematoxylin.

Double-labelling immunofluorescence was carried out on de-waxed sections, 4 μm-thick, which were stained with a saturated solution of Sudan black B (Merck, DE) for 15 min to block the autofluorescence of lipofuscin granules present in cell bodies, and then rinsed in 70 % ethanol and washed in distilled water. The sections were boiled in citrate buffer to enhance antigenicity and blocked for 30 min at room temperature with 10 % foetal bovine serum diluted in PBS. Then, the sections were incubated at 4 °C overnight with combinations of primary antibodies. After washing, the sections were incubated with Alexa488 or Alexa546 (1:400, Molecular Probes, US) fluorescence secondary antibodies against the corresponding host species. Nuclei were stained with DRAQ5™ (1:2,000, Biostatus, UK). After washing, the sections were mounted on Immuno-Fluore mounting medium (ICN Biomedicals, US), sealed, and dried overnight. Sections were examined with a Leica TCS-SL confocal microscope. Again, omission of the primary antibody in some sections was used as a control for the immunostaining.

Quantitative studies were carried out in the substantia nigra on serial non-consecutive sections stained with haematoxylin and eosin or processed for NPM1 and NPM3 immunohistochemistry. Nucleolar counts were performed directly under the ocular of the microscope at a magnification x200 in three areas (0.48 mm^2^) selected at random in every one of the eleven cases (2 PD1, 3 PD3, 3 PD4, 3 PD5). Results of NPM1 and NPM3-stained nucleoli per section were expressed as percentage of the total nucleoli visualized in haematoxylin and eosin-stained. Sections of PD at stage 1, stained with haematoxylin and eosin, were used to quantify neurons in which the nucleolus was visualized in that section.

To learn whether reduced NPM1 immunoreactivity in substantia nigra dopaminergic neurons was linked to α-synuclein inclusions, double-labelling immunofluorescence and confocal microscopy using anti-NPM1 and anti-α-synuclein antibodies was used to analyze six cases (1 tissue section per case) of PD at stages 4–5.

Quantification of co-localization of α-synuclein and eIF3 in sections processed for double-labelling immunofluorescence and confocal microscopy was done by counting 31 α-synuclein immunoreactive neurons (*n* = 6 sections) from PD stages 4 and 5, and noting how many of these neurons contained eIF3 immunoreactive inclusions. Results of eIF3 co-localization were expressed as the percentage of neurons with α-synuclein inclusions containing eIF3 deposits.

### α-synuclein oligomeric species in total homogenate fractions

Brain samples (0.1 g) of substantia nigra pars compacta, frontal cortex area 8 and putamen from MA (*n* = 3 per group) and stage 5 PD cases (*n* = 3 per group) were homogenized in a glass homogenizer, in 750 μl of ice-cold PBS+ (sodium phosphate buffer pH 7.0, plus protease inhibitors), sonicated, and centrifuged at 2,700 g at 4 °C for 10 min. The pellet was discarded and the resulting supernatant was ultra-centrifuged at 133,000 g at 4 °C for one hour. The supernatant (S2) was kept as the PBS-soluble fraction. The resulting pellet was re-suspended in a solution of PBS, pH 7.0, containing 0.5 % sodium deoxycholate, 1 % Triton, and 0.1 % SDS, and this was ultra-centrifuged at 133,000 g at 4 °C for one hour. The resulting supernatant (S3) was kept as the deoxycholate-soluble fraction. The corresponding pellet was re-suspended in a solution of 2 % SDS in PBS and maintained at room temperature for 30 min. Afterwards, the samples were centrifuged at 133,000 g at 25 °C for one hour and the resulting supernatant (S4) was the SDS-soluble fraction. Equal amounts of each fraction were mixed with reducing sample buffer and processed in parallel for 10 % SDS-PAGE electrophoresis and western blotting. Membranes were incubated with anti-α-synuclein oligomer-specific antibody (Agrisera, Vännäs, Sweden) at a dilution of 1:1,000. The protein bands were visualized with the ECL method (Amersham).

### α-synuclein oligomeric species in isolated nuclei

Small pieces of frozen brain samples (0.2 g) of frontal cortex area 8 from MA (*n* = 2, cases 4 and 15 of the Additional file 1: Table S1) and 2 PD cases stages 5–6 (*n* = 2, cases 113 and 120 of the Additional file 1: Table S1) were homogenized in a 6 ml ice-cold Solution D buffer (0.25 M sucrose, 25 mM potassium chloride (KCl), 5 mM magnesium chloride (MgCl_2_), and 20 mM Tris–HCl pH 7.5) with 0.1 % Triton, and then centrifuged at 1,000 g for 10 minutes at 4 °C (Ultracentrifuge Beckman with 70Ti rotor). The supernatant obtained was discarded and the pellet was re-suspended in 2 ml of Optiprep (D1556, Sigma, St Louis, MO, USA) to allow better separation by density gradient, and centrifuged at 3,200 g for 20 minutes at 4 °C. The new supernatant obtained was discarded again whereas the pellet was re-suspended in 500 μl ice-cold PBS buffer. Isolated nuclei were stained with mouse antibody to NeuN (see Additional file 3: Table S3). Primary antibody was visualized with appropriate secondary antibodies conjugated with Alexa 488. DNA content was determined using DAPI (4',6-diamidino-2-phenylindole). Subsequently, samples were centrifuged at 1,000 g for 10 min at 4 °C, and the solution obtained was re-suspended in 800 μl ice-cold PBS buffer.

Flow cytometry sorting was performed with a Beckman Coulter MoFlo Astrios. Nuclei were sorted at 25 PSI (pounds per square inch) through a 100 micron nozzle. Sample and collection tubes were kept at 10 °C for the duration of the sorting. Afterwards, NeuN+ (neuronal nuclei) and NeuN- (assumed glial nuclei) were collected separately in Optiprep and centrifuged at 3,200 g for 20 minutes at 4 °C. The pellet obtained was mixed with Laemmli buffer (2 % SDS, 10 % Glycerol, 0.002 % bromophenol blue, 6.25 mM Tris–HCl pH 6.8, bidistilled H_2_O, 2 % β-mercaptoethanol and phosphatase inhibitor cocktails), and pellets containing NeuN+ and NeuN- nuclei were processed in parallel for 10 % SDS-PAGE electrophoresis and western blotting. Demonstration that this fraction contained only nuclei without cytoplasmic contamination was carried out by western blotting with SOD-1 and histone H3 antibodies as indicated in Additional file 3: Table S3. Anti-α-synuclein oligomer-specific antibody (Agrisera, Vännäs, Sweden) was used to identify the presence of oligomers; the bands were visualized with the ECL method (Amersham).

## Results

### mRNA expression levels of nucleolar proteins in the substantia nigra, frontal cortex area 8, angular gyrus, precuneus, and putamen

Since nucleolar proteins are implicated in rRNA processing, the first step in the study was to analyse the mRNA expression levels of two chaperones and one protein linked to RNA polymerase.

**Fig. 1:**
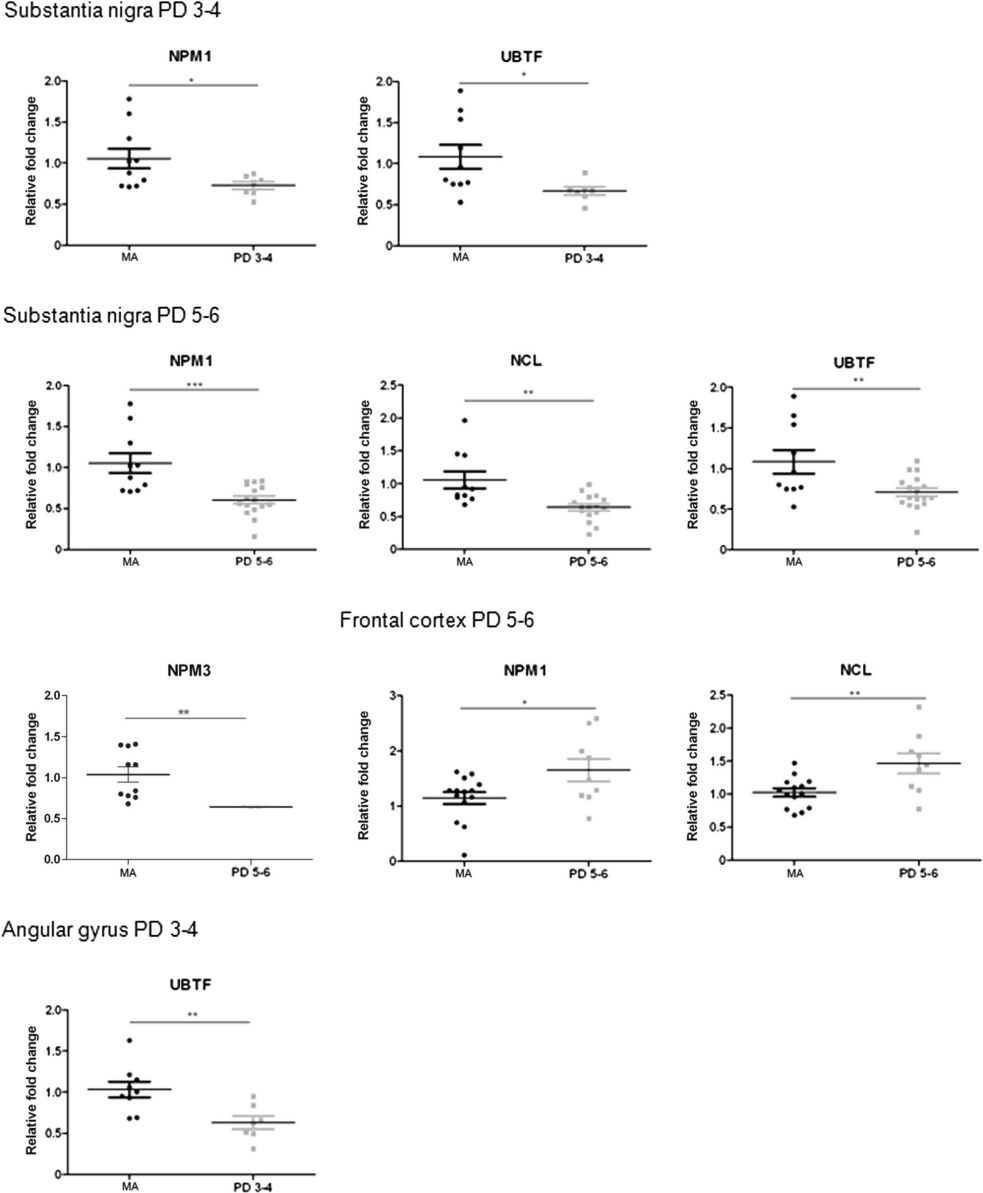
Altered mRNA expression levels of nucleolar proteins nucleophosmin (*NPM1/B23*), nucleoplasmin 3 (*NPM3*), nucleolin (*NCL*), and upstream binding transcription factor (*UBTF*) in the substantia nigra, frontal cortex area 8, and angular gyrus in middle-aged (MA) and PD as determined by TaqMan PCR assays using GUS-β for normalization. Reduced expression of *NPM1* and *UBTF* mRNAs is found in the substantia nigra at stages 3–4, but reduced *NPM1*, *NPM3*, *NCL,* and *UBTF* expression levels are found at stages 5–6. Only *NPM1* and *NCL* mRNAs are decreased in the frontal cortex at advanced stages of the disease. *UBTF* gene expression is transiently decreased at stages 3–4 in the angular gyrus. Student’s t test *p < 0.05, **p < 0.01 and ***p < 0.001

No modification in the mRNA expression levels of nucleophosmin (*NPM1*), nucleoplasmin 3 (*NPM3*), nucleolin (*NCL*), or upstream binding transcription factor (*UBTF*) was observed in the substantia nigra at PD stages 1–2 when compared with the MA group. However, *NPM1* and *UBTF* were significantly down-regulated (*p* < 0.05) at stages 3–4, as were *NPM1*, *NPM3, UBTF,* and *NCL* at stages 5–6 (*p* < 0.001) (Fig. 1). No significant differences were observed when comparing PD 3–4 with PD 5–6. Therefore, changes with disease progression were seen between stages 1–2 and stages 3–6 (see Additional file 4: Table S4).

No differences in *NCL*, *NPM1, NPM3,* or *UBTF* mRNA expression were observed in frontal cortex area 8 at stages 3–4 when compared with the MA group, but *NPM1* and *NCL* mRNAs were significantly increased (*p* < 0.05, and *p* < 0.001, respectively) at stages 5–6 (Fig. 1).

A transient decrease in *UBTF* mRNA expression was found in the angular gyrus at stages 3–4 (Fig. 1). No modifications in the expression of *NPM1*, *NCL, NPM3,* or *UBTF* mRNAs were identified in the precuneus and putamen at any stage analysed.

Details of all genes analyzed are found in Additional files 4, 5, 6, 7 and 8.

### 18S rRNA and 28S rRNA in the substantia nigra, frontal cortex area 8, angular gyrus, precuneus, and putamen

**Fig. 2:**
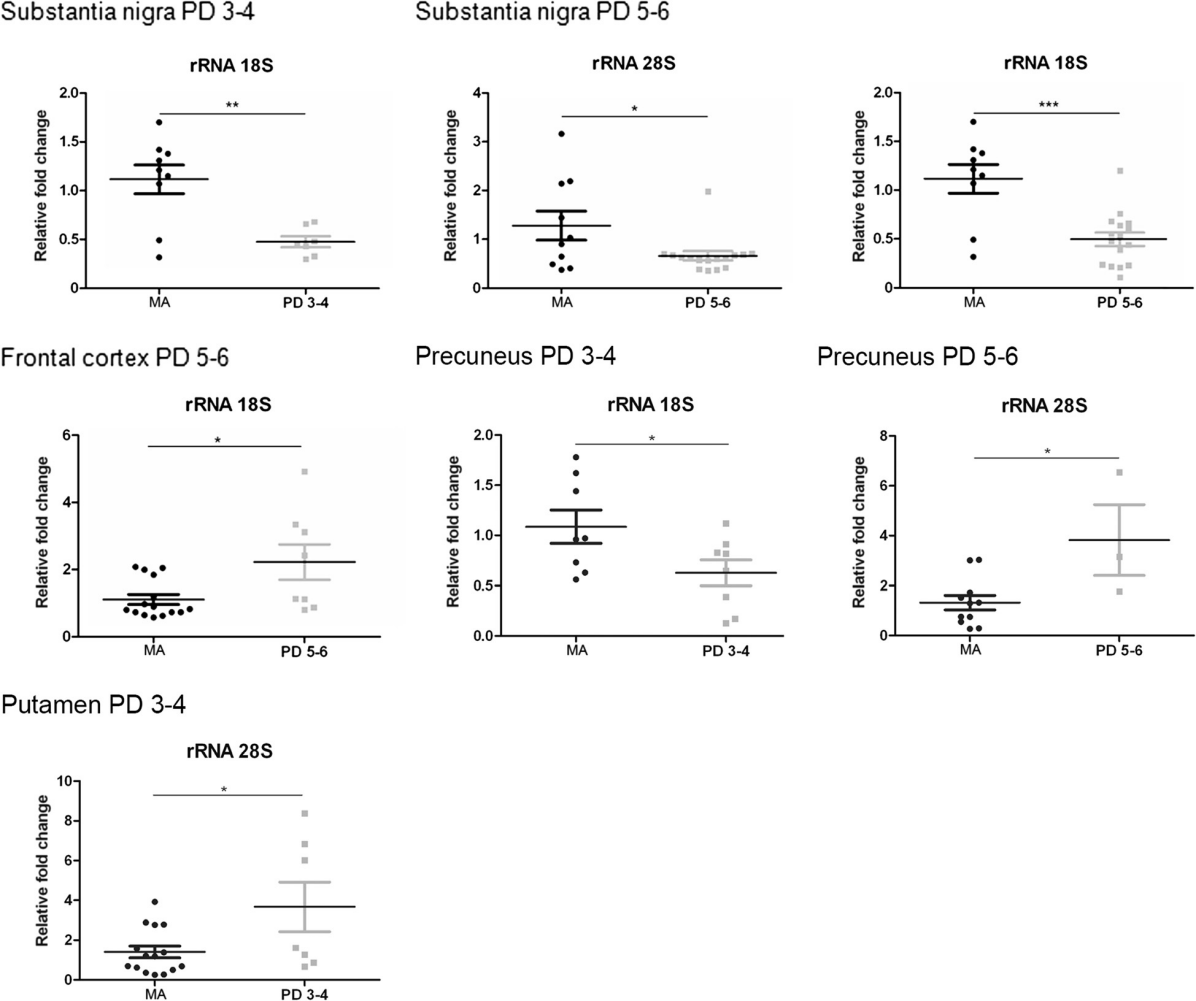
Altered expression levels of rRNA28S and rRNA18S in substantia nigra, frontal cortex area 8, angular gyrus, precuneus, and putamen in middle-aged (MA) and PD as determined by TaqMan PCR assays using GUS-β for normalization. Student’s t test *p < 0.05, **p < 0.01 and ***p < 0.001

In the substantia nigra, 18S rRNA levels were significantly reduced at stages 3–4 (*p* < 0.01), as were 28S rRNA and 18S rRNA levels at stages 5–6 (*p* < 0.05 and *p* < 0.001, respectively) (Fig. 2). Therefore, differences along disease progression were seen between stages 1–2 and 3–6 (Additional file 4: Table S4).

In frontal cortex area 8, a significant increase in 18S rRNA (*p* < 0.05) was found at stages 5–6 (Fig. 2). rRNA expression was not altered in the angular gyrus. A transient 18S rRNA decrease (*p* < 0.05) and a transient 28S rRNA increase (*p* < 0.05) was noted in the precuneus and putamen, respectively, at stages 3–4. 28S rRNA up-regulation (*p* < 0.05) was observed in the precuneus at stages 5–6 (Fig. 2). Therefore, definite up-regulation of 18S rRNA and 28S rRNA was identified in the frontal cortex and precuneus, respectively, at advances stages of PD.

Details of rRNA results in all regions and stages are found in Additional files 4, 5, 6, 7 and 8.

### mRNA expression levels of genes encoding ribosomal proteins in the substantia nigra

Since ribosomal proteins are essential to the assembly of ribosomal subunits and to the process of protein synthesis, the next step was to analyse gene expression of 9 RPL and 7 RPS genes. Selection of these mRNAs was done at random.

**Fig. 3:**
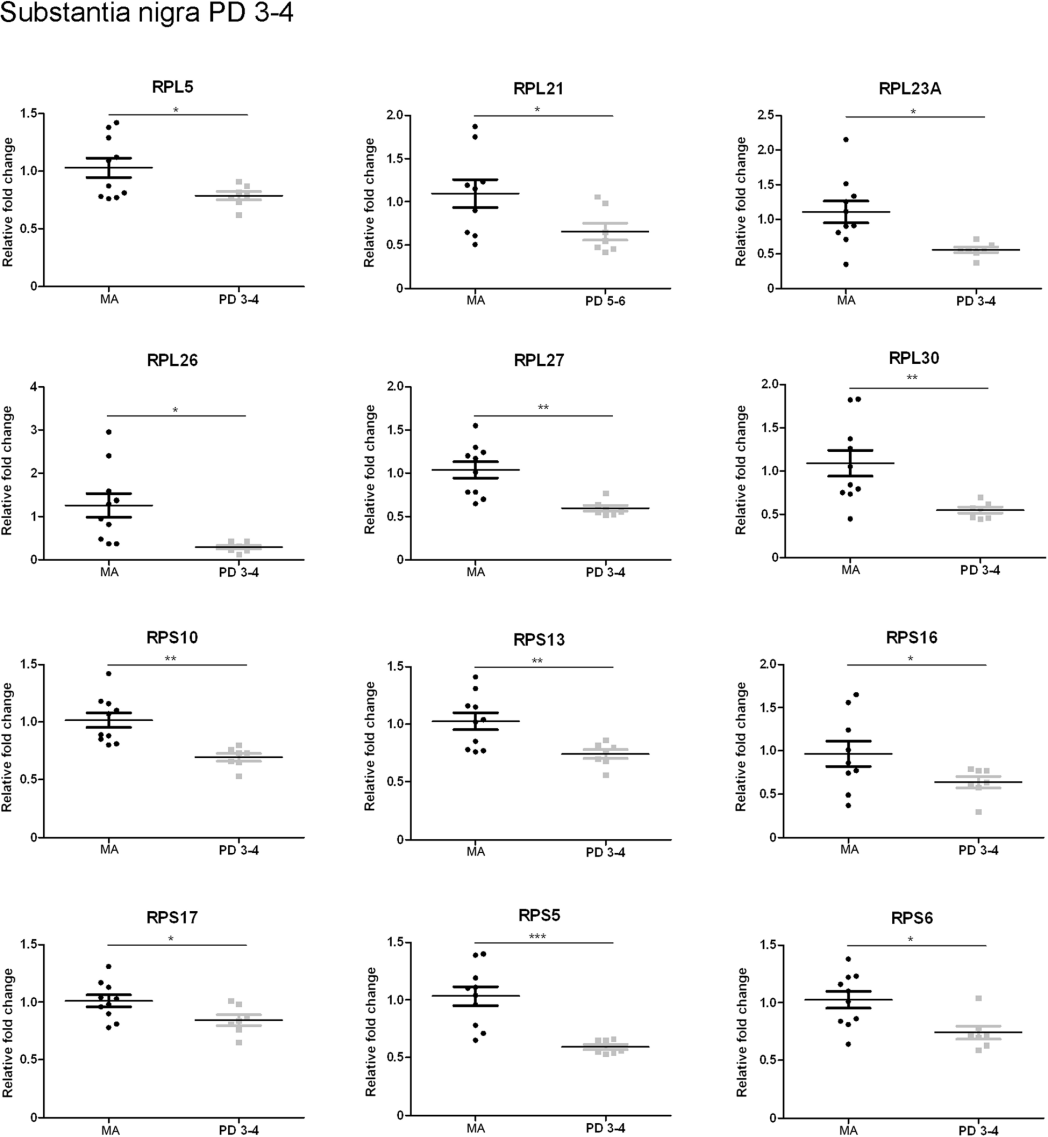
Altered mRNA expression levels of 16 ribosomal proteins in the substantia nigra in middle-aged (MA) and PD cases determined by TaqMan PCR assays using GUS-β for normalization. Student’s t test *p < 0.05, **p < 0.01 and ***p < 0.001

**Fig. 4:**
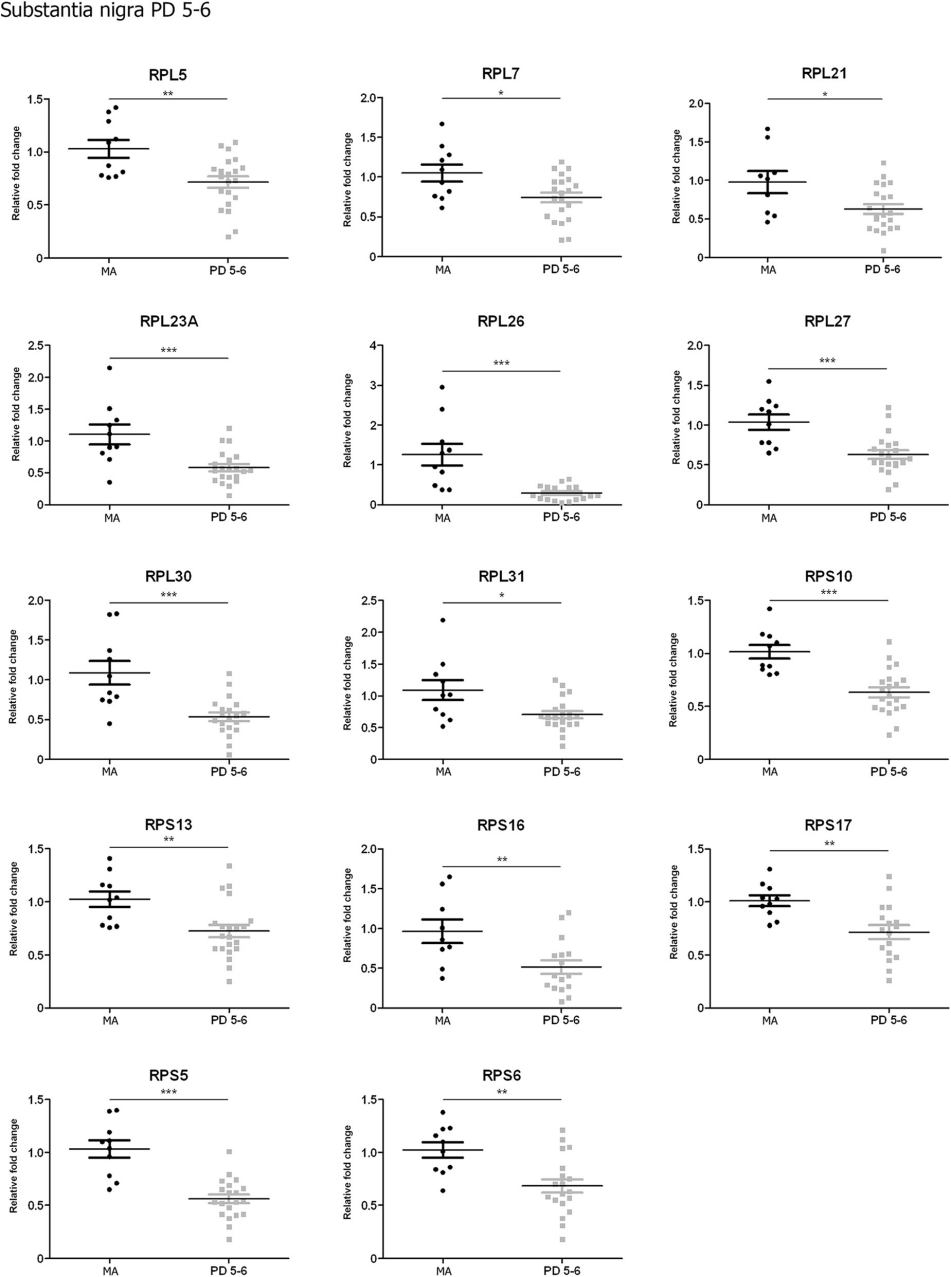
Altered mRNA expression levels of 16 ribosomal proteins in the frontal cortex area 8 in middle-aged (MA) and PD cases determined by TaqMan PCR assays using GUS-β for normalization. Student’s t test *p < 0.05, **p < 0.01 and ***p < 0.001

No significant changes were observed in the substantia nigra at stages 1–2. However, twelve of sixteen genes analysed were significantly down-regulated in the substantia nigra at stages 3–4 including *RPL5*, *RPL21*, *RPL23A*, *RPL26*, *RPL27*, *RPL30*, *RPS10*, *RPS13*, *RPS16*, *RPS17*, *RPS5,* and *RPS6* (p values varied from < 0.05 to <0.001) (Fig. 3). Fourteen genes were down-regulated in the substantia at stages 5–6. These included, in addition to those down-regulated at stages 3–4, *RPL7* and *RPL31* (p values ranged from < 0.01 to < 0.001) (Fig. 4). Therefore, major modifications with disease progression were found between stages 1–2 and stages 3–6.

Details of all genes analyzed are found in Additional file 4: Table S4.

### mRNA expression levels of genes encoding ribosomal proteins in frontal cortex area 8, angular gyrus, precuneus, and putamen

**Fig. 5:**
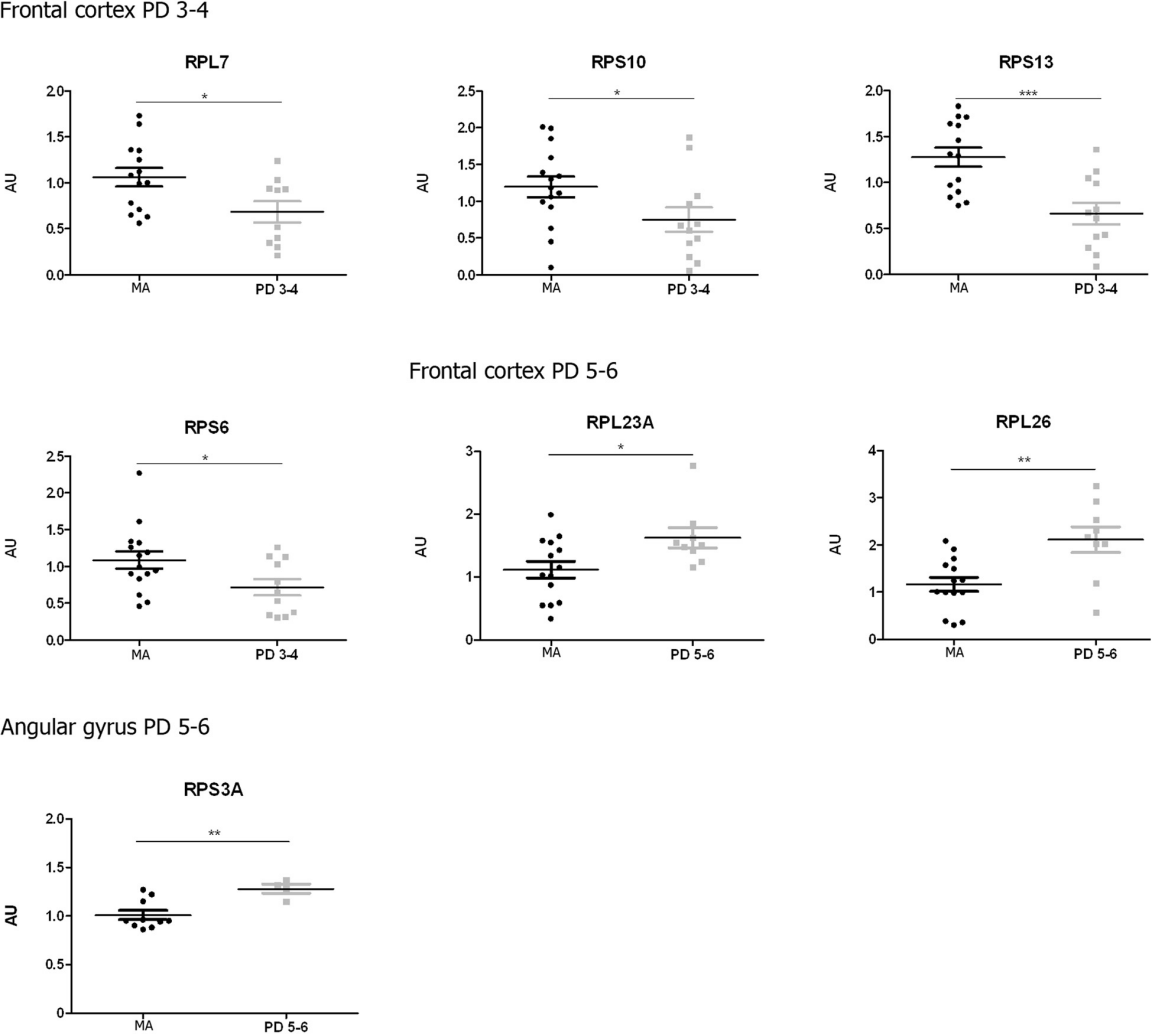
Altered mRNA expression levels of 16 ribosomal proteins in the angular gyrus in middle-aged (MA) and PD cases determined by TaqMan PCR assays using GUS-β for normalization. Student’s t test *p < 0.05, **p < 0.01 and ***p < 0.001

A transient down-regulation in the expression of *RPL7* (*p* < 0.05), *RPS6* (*p* < 0.05), *RPS10* (*p* < 0.05) and *RPS13* (*p* < 0.001) was found in frontal cortex area 8 at stages 3–4. However, two genes were up-regulated in the frontal cortex at stages 5–6: *RPL23A* (*p* < 0.05) and *RPL26* (*p* < 0.01) (Fig. 5). In short, five genes were significantly up-regulated in the frontal cortex when comparing PD stages 3–4 and PD stages 5–6: *RPL7*, *RPL22*, *RPL23A*, RPL26, *RPS6,* and *RPS17* (see Additional file 5: Table S5).

Only one gene, *RPS3A,* was up-regulated (*p* < 0.01) in the angular gyrus at stages 5–6 (Fig. 5), thus indicating modifications with disease progression (see Additional file 6: Table S6).

**Fig. 6:**
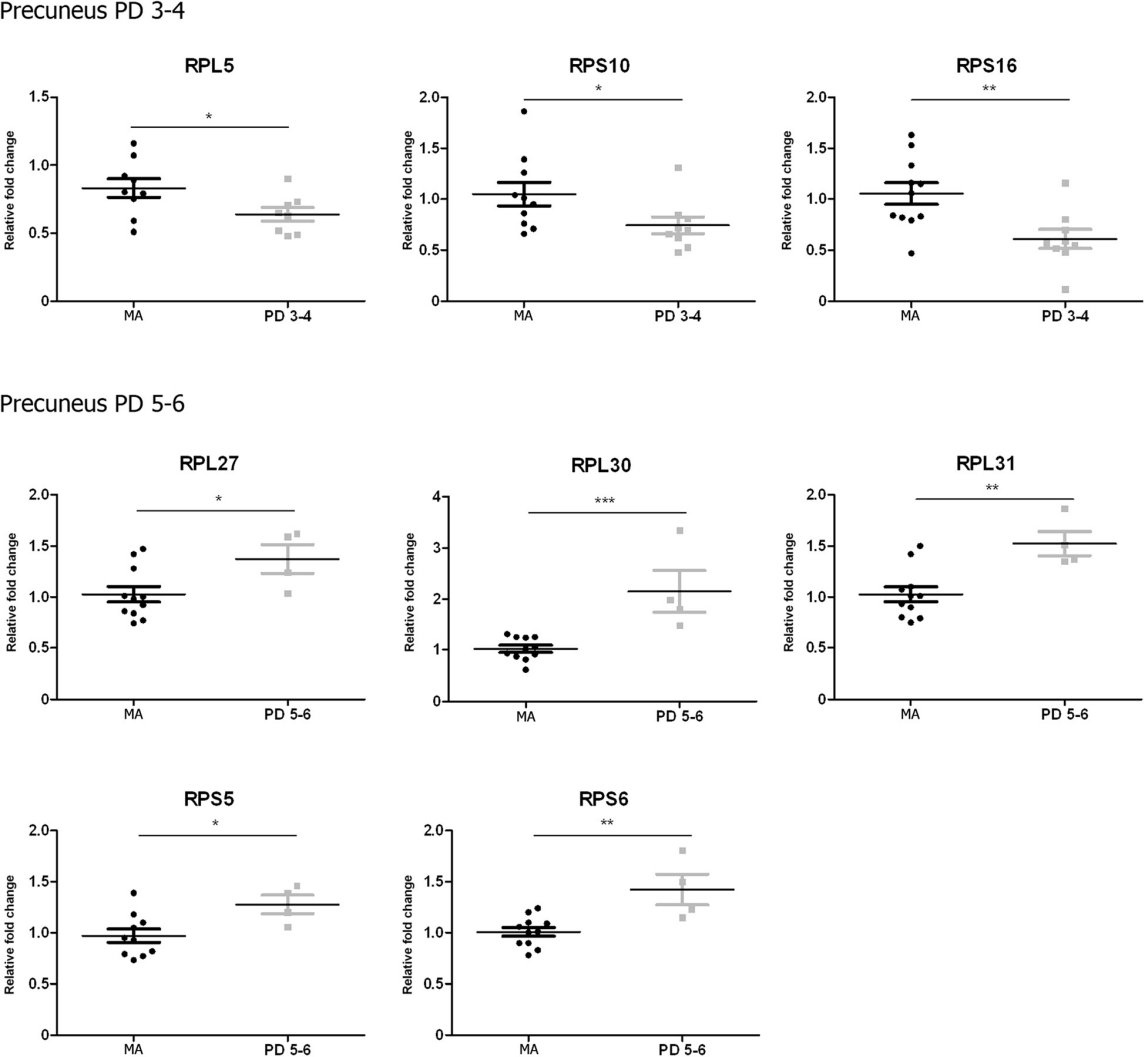
Altered mRNA expression levels of 16 ribosomal proteins in the precuneus in middle-aged (MA) and PD cases determined by TaqMan PCR assays using GUS-β for normalization. Student’s t test *p < 0.05, **p < 0.01 and ***p < 0.001

The precuneus showed a similar pattern to frontal cortex area 8 with decreased mRNA expression of certain genes at PD stages 3–4 followed by increased expression of other genes at stages 5–6. Yet the genes involved differed in the frontal cortex area 8 from the precuneus. Reduced *RPL5* (*p* < 0.05), *RPS10* (*p* < 0.05), and *RPS16* (*p* < 0.01) mRNA expression occurred at stages 3–4 compared to the MA group. Increased *RPL27* (*p* < 0.05), *RPL30* (*p* < 0.001), *RPL31* (*p* < 0.01), *RPS5* (*p* < 0.05), and *RPS6* (*p* < 0.01) mRNA expression was found at stages 5–6 (Fig. 6).

Only *RPL5* of the sixteen assessed RNAs encoding ribosomal proteins was increased at stages 5–6 in the putamen in PD (see Additional file 8: Table S8).

### Immunohistochemistry of nucleolar proteins in the substantia nigra

To learn whether mRNA changes in nucleolar chaperones translated into altered protein expression, immunohistochemistry and immunofluorescence to NPM1 and NPM3 were performed in the substantia nigra.

A similar approach was tried for the study of ribosomal proteins. After testing more than twelve antibodies, none of them was useful for immunohistochemistry and western blotting.

**Fig. 7:**
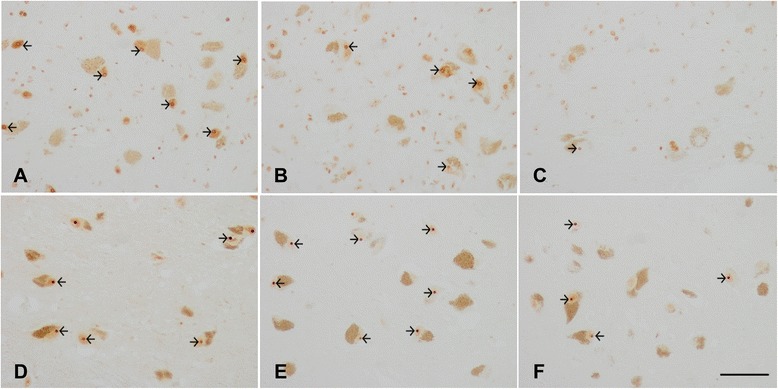
**a**-**c**: Nucleophosmin (NPM1) immunohistochemistry in the substantia nigra pars compacta in PD at stages 1–2 (**a**), 3–4 (**b**), and 5–6 (**c**) showing NPM1 immunoreactivity in the nucleoli (arrows). The number and intensity of NPM1 immunoreactivity decreases at advanced stages of PD. **d**-**f**: Nucleoplasmin 3 (NPM3) immunohistochemistry in the substantia nigra pars compacta in PD at stages 1–2 (**d**), 3–4 (**e**), and 5–6 (**f**) showing preserved NPM3 immunoreactivity in the nucleolus (arrows). Paraffin section without haematoxylin counterstaining, bar = 50 μm

NPM1 and NMP3 localized in the nucleolus in MA and diseased cases. However, decreased NPM1 immunoreactivity was reduced in pigmented neurons (Fig. 7a-c). In contrast, NPM3 immunoreactivity was preserved in the majority of neurons (Fig. 7d-f).

Quantitative studies were performed to rule out reduced or missing NPM1 immunoreactivity being due to artefacts of nucleolar sectioning or to altered nucleolar immunoreactivity to different nucleolar markers. Counts were made on serial non-consecutive sections of the same cases stained with haematoxylin and eosin, and processed for NPM1 and NPM3 immunohistochemistry without counterstaining. The percentage of neurons in the substantia nigra pars compacta with visualized nucleoli at PD stage 1, as assessed in haematoxylin and eosin sections, and NPM1 and NPM3 immunohistochemistry, was about 50 %. The total number of neurons decreased with disease progression, representing about 40 % neuron loss at stage 5 when compared with PD stage 1. NPM3-immunoreactive nucleoli were found in 44 % of remaining neurons at stage 5, but the percentage of NPM1-immunoreactive nucleoli in remaining neurons was around 34 % at stage 5. Weakly stained nuclei were considered positive nuclei. Quantitative results are shown in Additional file 9: Table S9.

These results suggest that reduced NPM1 immunoreactivity is not merely a reflection of neuron loss but rather indicates selective vulnerability of NPM1 when compared with NPM3 to PD.

Whether decreased NPM1-immunoreactive nucleoli in advanced stages of PD was a reflection of the co-occurrence of Lewy bodies in a particular neuron was assessed using double-labelling immunofluorescence and confocal microscopy with anti-NPM1 and anti-α-synuclein antibodies in six cases (1 tissue section per case) of PD at stages 4–5. Decreased nucleolar NPM1 immunoreactivity occurred independently of the presence or absence of α-synuclein inclusions in the cytoplasm of neurons (data not shown).

### Protein expression of initiation and elongation transcription factors in substantia nigra and frontal cortex area 8

The expression levels of initiation factors eIF1, eIF2-α, P-eIF2-α, eIF3, eIF3η, and eIF5, and elongation factors eEF1A and eEF2, were analysed with western blotting. These antibodies did not work for immunohistochemistry.

**Fig. 8:**
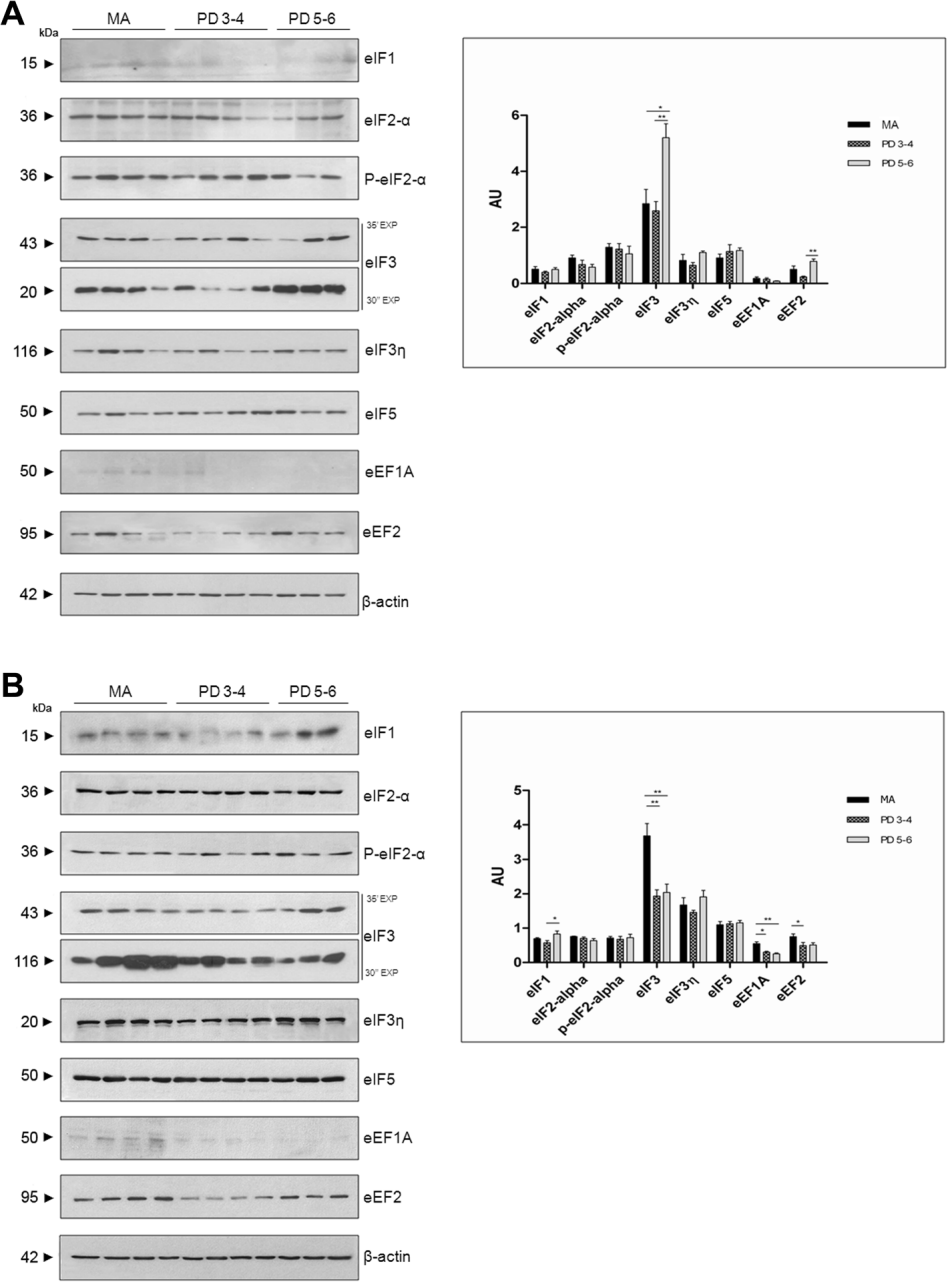
Western blotting of eukaryotic initiation factors eIF1, eIF2-α, eIF3, eIF3 , and eIF5, and elongation factors eF1A and eF2 in the substantia nigra (**a**) and frontal cortex area 8 (**b**). Significantly increased expression of eIF3 and eEF2, more marked at stages 5–6, is observed in the substantia nigra in PD compared with MA individuals. In contrast, eIF3 expression is significantly reduced in frontal cortex area 8 at stages 3–4 and 5–6, whereas eIF1 is significantly increased at stage 5–6. Expression of elongation factors eF1A and eF2 is also significantly reduced in the frontal cortex in PD; β-actin is used as a control of protein loading. AU:arbitrary units. *p < 0.05; **p < 0.01

A significant increase in eIF3 and eEF2 was observed in the substantia nigra, especially at stages 5–6, when compared with the values for the MA group (Fig. 8a).

In contrast, eIF3 expression levels were significantly reduced at stages 3–4 (*p* < 0.01) and 5–6 (*p* < 0.001), whereas eIF1 expression levels were significantly increased (*p* < 0.05) at stages 5–6 in frontal cortex area 8. Regarding elongation factors, eEF1A and eEF2 protein expression was significantly decreased at stages 3–4 (*p* < 0.05), and more markedly so for eEF1A (*p* < 0.01) at stages 5–6 (Fig. 8b).

### Reticulum stress markers in substantia nigra and frontal cortex area 8

Abnormal protein synthesis and accumulation of abnormal proteins in the endoplasmic reticulum is causative of the endoplasmic reticulum stress response [18]. For this reason, we also explored the unfolded protein response (UPR) in the two brain areas in PD.

The expression levels of proteins GRP78, GRP94, ATF4, ATF6, XBP1, p54, and P-IRE-1 were analysed in the substantia nigra and frontal cortex area 8 in PD cases at stages 3–4 and 5–6 in comparison with samples from MA individuals.

**Fig. 9:**
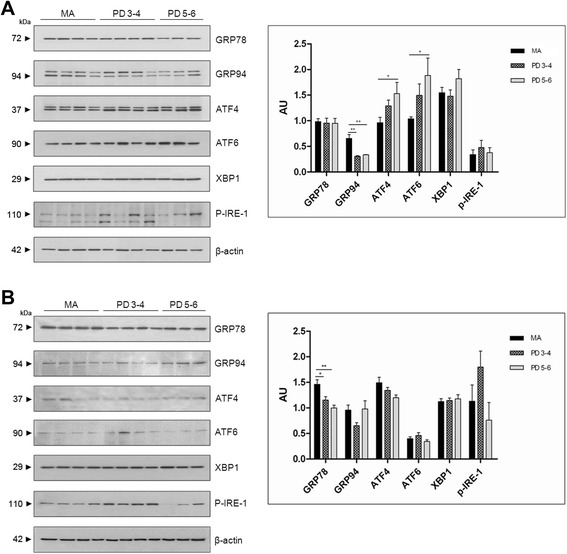
Western blotting of glucose-regulated protein 78 (GRP78), glucose-regulated protein 94 (GRP94), activating transcription factor 4 (ATF4), activating transcription factor 6 (ATF6), Xbox binding protein 1 (XBP1), and phosphorylated inositol requiring kinase 1 (P-IRE-1) in the substantia nigra (**a**) and frontal cortex area 8 (**b**) shows significantly increased expression of ATF4 and ATF6 90 kDa at stages 5–6 (*p* < 0.05), and reduced expression of GRP94 at stages 3–4 and 5–6 (*p* < 0.01) in the substantia nigra in PD when compared with MA individuals. ATF6f (50 kDa), the cleaved and active form of ATF6, was not identified in any group. In contrast, significant reduction of GRP78 (*p* < 0.01 at stages 3–4, and *p* < 0.001 at stages 5–6) with preservation of other reticulum stress markers was found in frontal cortex area 8 in PD. Note that a double band with GRP94, ATF4, and P-IRE-1 antibodies is observed in the substantia nigra but not in the frontal cortex. The expected molecular weight is marked by an arrowhead in SN western blots. β-actin is used as a control of protein loading. AU: arbitrary units; *p < 0.05; **p <0.01

Increased expression levels of ATF4 and ATF6 90 kDa at stages 5–6 (*p* < 0.05), and reduced GRP94 expression levels at stages 3–4 and 5–6 (*p* < 0.01), were found in the substantia nigra in PD (Fig. 9a). No ATF6f (50 kDa) was identified in any group (data not shown). Reduced expression levels of GRP78 were observed in frontal cortex area 8 at stages 3–4 and 5–6 (*p* < 0.01 and *p* < 0.001, respectively). No changes in the expression of other reticulum stress markers were found in frontal cortex area 8 in PD (Fig. 9b).

**Fig. 10:**
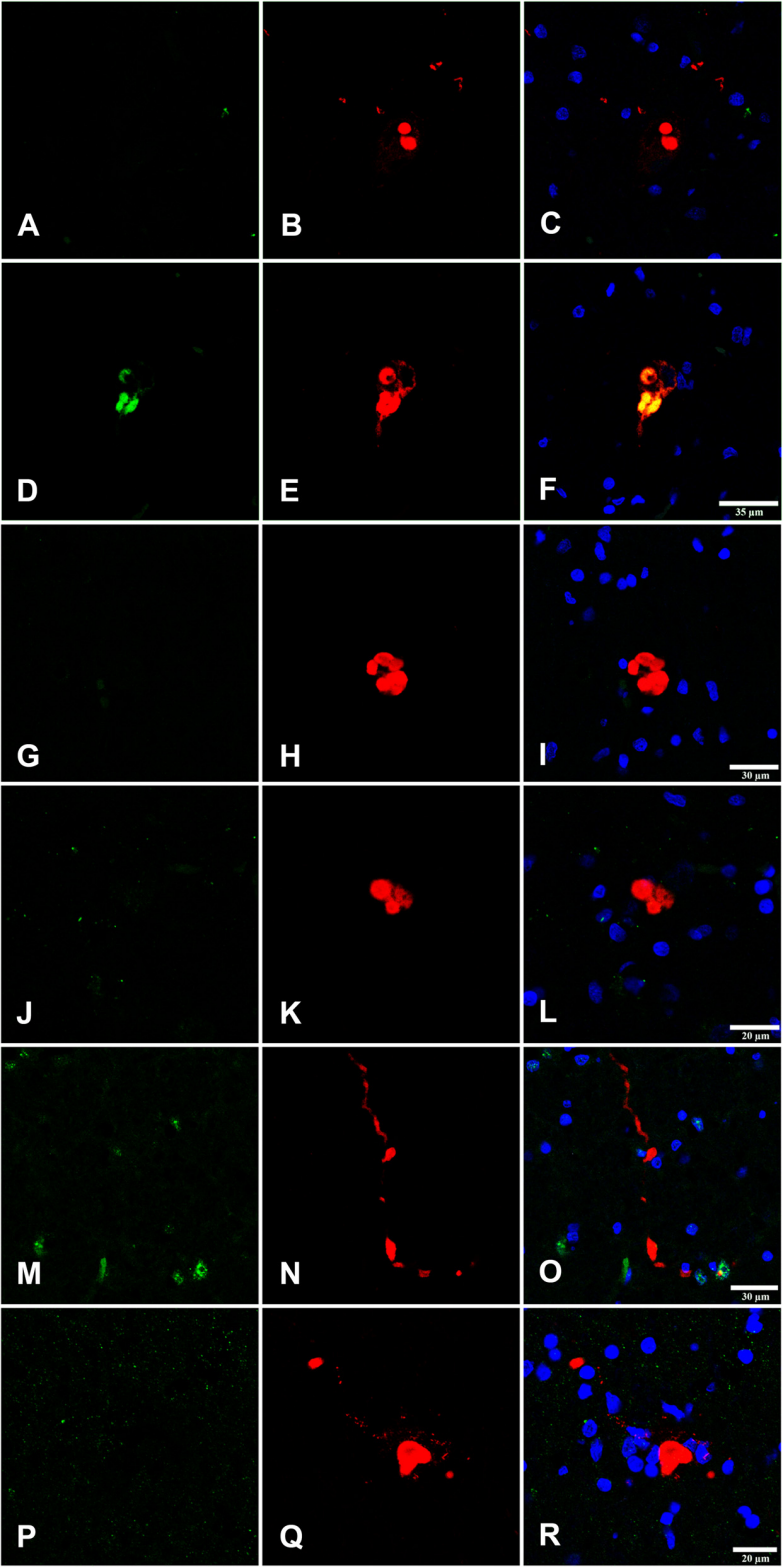
Double-labelling immunofluorescence and confocal microscopy in the substantia nigra in PD cases stages 4 and 5 to α-synuclein (**b**, **e**, **h**, **k**, **n**, **q**) and p54 (**a**), eIF3 (**d**), GRP78 (**g**), GRP94 (**j**), IRE1 (**m**), and XBP1 (**p**); **c**, **f**, **i**, **l**, **o**, **r**: merge. α-synuclein in Lewy bodies and neurites (red) only co-localizes with eIF3 (green). Paraffin sections, nuclei are stained with DRAQ5^TM^. **a**-**f**, bar = 35 μm; G-I, m-o, bar = 30 μm; **j**-**l**, **p**-**r**, bar = 20 μm

Double-labelling immunofluorescence and confocal microscopy in the substantia nigra in PD cases stages 4 and 5 showed α-synuclein in Lewy bodies and neurites co-localizing with eIF3. Quantitative studies showed 27 of 31 α-synuclein immunoreactive neurons co-localizing with eIF3 in Lewy bodies (87 %). In contrast, GRP78, GRP94, IRE-1, and XBP1 did not show co-localization with α-synuclein inclusions (Fig. 10).

### α-synuclein oligomeric species in total homogenate fractions

The anti-α-synuclein oligomer-specific antibody stained Lewy bodies and neurites, and small granules in substantia nigra neurons in paraffin sections. The background was clear, and the small dots consistent with small deposits of oligomers in the neuropil were extremely rare (data not shown).

**Fig. 11:**
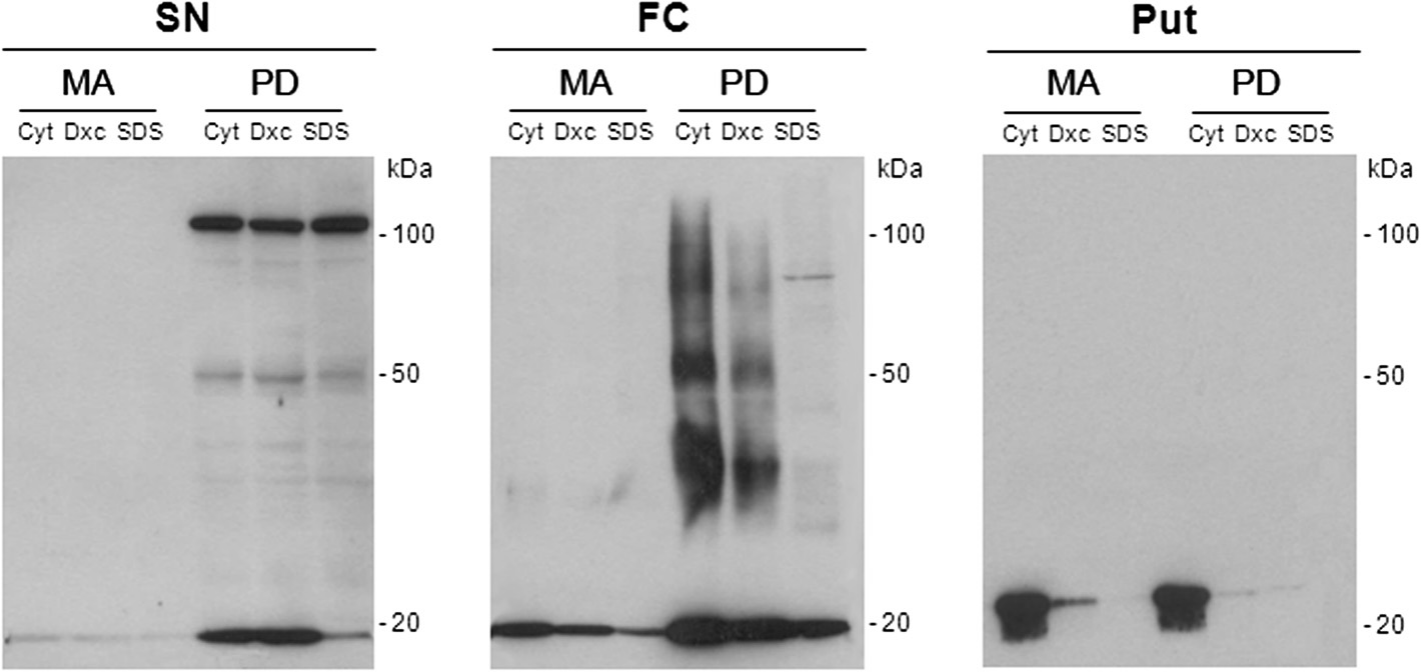
Western blotting of α-synuclein oligomeric species in the substantia nigra pars compacta (SN), frontal cortex area 8 (FC), and putamen (Put) in total homogenate cytosolic (Cyt), deoxycholate (Dxc), and sodium dodecyl sulphate (SDS) fractions in middle-aged individuals (MA) and Parkinson’s disease stages 5–6 (PD). α-synuclein oligomeric species are only observed in the three fractions in PD but not in MA cases, thus indicating abnormal α-synuclein solubility and aggregation of α-synuclein in the substantia nigra and frontal cortex in PD. However, the patterns of α-synuclein oligomers differ in the three regions. Two net bands of 50 kDa and about 100 kDa are seen in the three fractions in the substantia nigra, but three bands with a considerable smear of about 30 kDa, 50 kDa, and 90 kDa are found, mainly in the cytosolic and deoxycholate fractions, in the frontal cortex. In addition, note the higher density of α-synuclein bands in the frontal cortex when compared with the substantia nigra in PD. No α-synuclein oligomers are detected in the putamen, whereas a band of about 17 kDa is found in MA and PD cases

The biochemical analysis of α-synuclein oligomeric species was carried out on frozen samples of the substantia nigra*,* frontal cortex area 8, and putamen in MA and PD cases in cytosolic (PBS; Cyt), deoxycholate (Dxc), and sodium dodecyl sulfate (SDS) fractions. In the substantia nigra, a band of α-synuclein at the expected molecular weight, about 17 kDa, was observed in MA and PD cases at stages 5–6 in the three fractions, although the density of the band was higher in PD than in MA cases. In addition, two well-defined bands of 50 kDa and approximately 100 kDa were obtained in all three fractions (Cyt, Dxc, and SDS) in the substantia nigra in PD (Fig. 8). α-synuclein oligomeric species were also seen in the frontal cortex in PD at stages 5–6, but the band pattern differed from that seen in substantia nigra. In addition to the band of about 17 kDa found in MA and PD cases, three bands of molecular weight of about 35 kDa, 50 kDa, and 90 kDa, all of them with marked smear, were observed mainly in the cytosolic and deoxycholate fractions in the frontal cortex only in PD (Fig. 8). In contrast, no oligomeric species were detected in the putamen; only the band of about 17 kDa was identified equally in MA and PD cases (Fig. 11).

### α-synuclein oligomeric species in isolated nuclei obtained by FACS

**Fig. 12:**
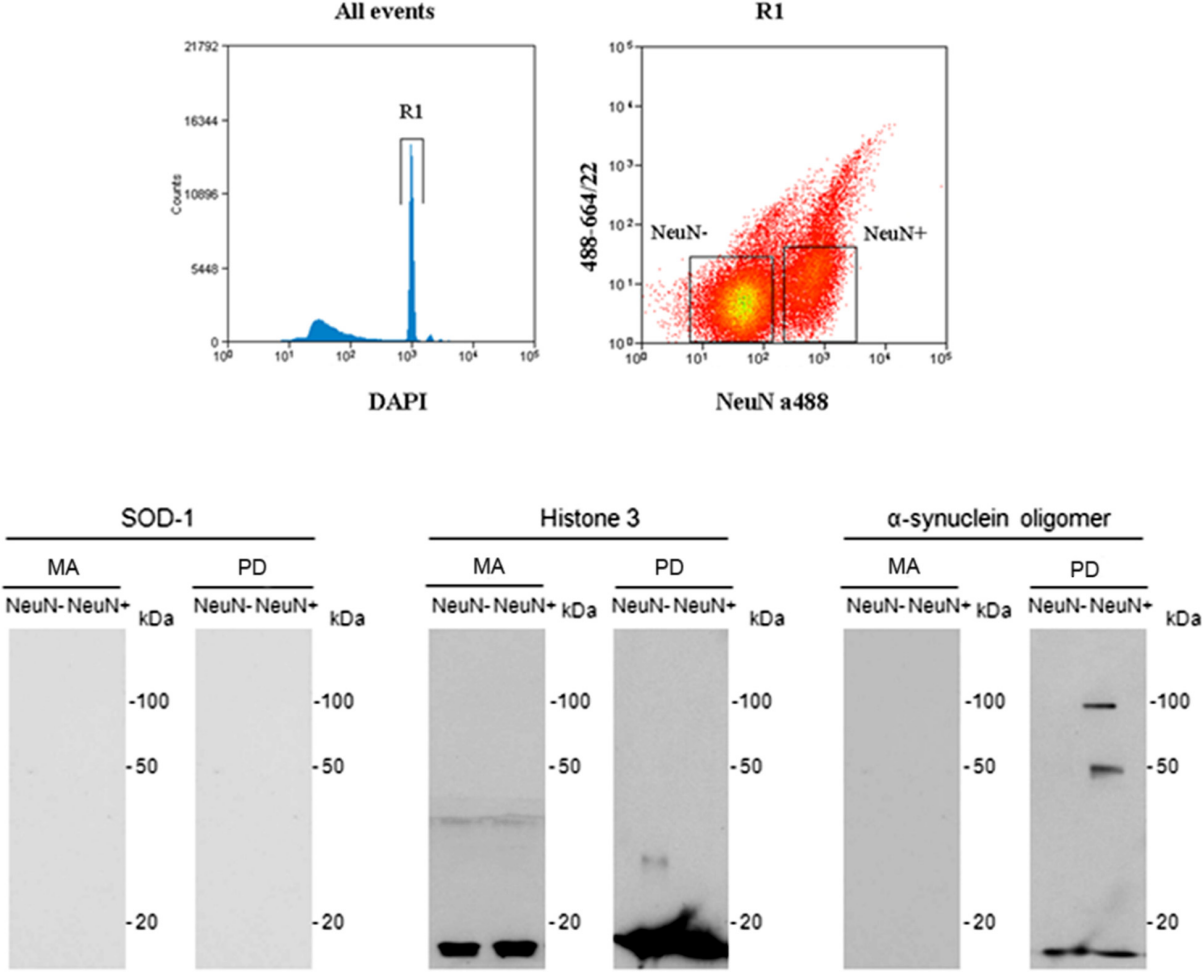
Illustrating example of the gating strategy used for neuronal nuclei sorting. Single nuclei were gated (R1) based on their DAPI labelling, followed by gating neuronal nuclei (NeuN+) and non-neuronal nuclei (NeuN-) on the NeuN vs red autofluorescence dot plot. Representative western blotting of isolated nuclei obtained by FACS of frontal cortex area 8 of middle-aged (MA) and Parkinson’s disease (PD) stage 5–6 cases. No signal was detected with anti-superoxide dismutase 1 (SOD-1) antibodies but there was one specific band at 17 kDa with anti-histone H3 antibody, thus demonstrating no cytoplasmic contamination of isolated nuclei. One band at about 20 kDa and two bands at about 50 kDa and at 100 kDa are detected with specific anti-α-synuclein oligomeric species in the neuronal nuclear sample (NeuN+) in PD. Interestingly, a weak α-synuclein low band is also detected in the non-neuronal nuclei sample in PD

Nuclear fractions were obtained by FACS using DAPI and NeuN antibody from the grey matter of frontal cortex area 8 in MA and PD cases (Fig. 12a). DAPI binds strongly to DNA and labels all nuclei in the suspension regardless of cell type. Using this approach, it is not difficult to discriminate single nuclei (R1) from doublets, triplets, and aggregates, as well as debris and background noise. Nuclei selected in R1 were sorted into neuronal (NeuN+) and non-neuronal (NeuN-). To define more accurately the population, NeuN was analysed on the same plot as red fluorescence to eliminate cells not completely lysed, permeable to DAPI, and with more autofluorescence than isolated nuclei. Purity of the nuclear fractions was assessed by demonstrating histone H3 immunoreactivity (a band at 17 kDa) and lack of SOD-1 immunoreactivity in the isolated nuclei in both NeuN- and NeuN+ samples in MA and PD cases. Western blots using specific antibodies to α-synuclein oligomeric species demonstrated in the NeuN+ samples one band at about 20 kDa and two bands of about 50 kDa and 100 kDa in PD cases. One weak band at about 20 kDa was also seen in the NeuN- samples in PD (Fig. 12b). No bands of α-synuclein oligomers were detected in NeuN+ and NeuN- samples in MA cases processed in parallel, excepting weak bands after long exposure in some cases (Additional file 10: Figure S1).

## Discussion

Nucleolar proteins and rRNAs are differentially regulated in the *substantia nigra*, frontal cortex area 8, angular gyrus, precuneus, and putamen in PD, and changes accelerate with disease progression

Ribosomal RNA genes are arranged in tandem repeats in the NORs and are transcribed by RNA polymerase I in conjunction with associated factors including UBF (upstream binding transcription factor, encoded by *UBTF*) and SL1 proteins [19–26]. rDNAs encode precursor transcripts which are processed to form 18S, 28S, and 5.8S RNAs [27]. UBF is localized in the nucleoli in interphase cells and it regulates RNA polymerase 1 following acetylation [28–30].

Nucleolin (NCL) and nucleophosmin (NPM1/B23) are other major nucleolar proteins acting as histone-binding chaperones required for chromatin compaction, regulation of rRNA transcription, nucleic acid binding, and nuclear re-programming [10, 26, 31–42]. In addition, NPM1 is involved in the nuclear transport of proteins to the nucleolus and of certain ribosomal proteins to the cytoplasm [43–46]. NPM1-null mice display altered ribosome biogenesis and premature death at mid-gestation [47, 48]. NPM1 acts as a ribonuclease for the maturation of rRNA transcript [49, 50], silencing NPM1 results in altered processing of 28S RNA [51]. Additional functions of NPM1 are related to DNA replication, transcription, and repair [37]. NPM3 is a nucleolar histone chaperone that interacts with nucleophosmin and modulates ribosome biogenesis [52, 53].

Cellular and molecular alterations associated with impaired nucleolar activity are causative of nucleolar stress [54–56] which can lead to the malfunction of the nucleolar machinery, altered rRNA expression, reduced protein synthesis, and, when extreme, cell death. Nucleolar stress is emerging as an important sensor in several pathological conditions [57] including ischemic damage, cancer [58–60], and neurodegeneration [61–63]. Nucleolar stress has been documented in AD [64], and in polyglutamine diseases including Huntington’s disease and related models [65–68].

The present study identifies reduced *NCL* and *NPM1* mRNA levels and NPM1 immunoreactivity in the substantia nigra in PD cases with disease progression. *NPM3* mRNA expression levels are also reduced in the substantia nigra at advanced stages. Reduced mRNA expression may be related to the progressive loss of dopaminergic neurons in the substantia nigra, but preserved expression of NPM3 protein, in contrast to decreased NPM1 immunoreactivity in remaining dopaminergic neurons, indicates selective vulnerability of NPMs to neurodegeneration. Reduced *NCL* mRNA is in agreement with previous observations showing reduced nucleolin protein expression in the substantia nigra in PD [69]. The reduced *UBTF* here observed is also in line with previous observations of nucleolar disruption in dopaminergic neurons in PD [8]. The reduced nucleophosmin expression in the substantia nigra in PD here observed for the first time may act as an additional cause of neurodegeneration [70].

Reduced expression of 18S rRNA and 28S rRNA gives strong support to the concept that nucleolar stress is a major alteration in the substantia nigra in PD [64]. Although decreased biosynthesis of ribosome subunits may be a response to preserve energy homeostasis in acute stress situations [71], it is less clear that maintained reduced expression of rRNAs has any beneficial effect on cell survival. Rather, perpetuation of nucleolar stress and reduced rRNA synthesis is consistent with parallel cell damage in PD substantia nigra.

In contrast to the substantia nigra, *NCL* and *NPM1* mRNAs are increased in frontal cortex area 8 in PD at stages 5–6, whereas *UBTF* appears to be transiently decreased in the angular gyrus at stages 3–4. No modifications in *NCL*, *NPM1,* and *UBTF* gene expression are seen in the precuneus and putamen at any stage of the disease. In contrast to the substantia nigra, 18S rRNA and 28S rRNA are increased in the frontal cortex and precuneus, respectively, at stages 5–6 of Braak. Overexpression of NCL is neuroprotective against rotenone, a toxicant used to experimentally reproduce some characteristics of PD in animal models [72]. Overexpression of NPM1 is neuroprotective against kainic acid-induced excitotoxicity [73, 74]. Therefore, increased *NCL* and *NPM1* mRNA expression in frontal cortex appears to be a response to PD geared to protecting rRNA synthesis. According to this hypothesis, 18S rRNA expression is increased in frontal cortex at advanced stages of PD. Lack of correlation between preserved *NCL* and *NPM1* expression and increased 28S rRNA in the precuneus may be related to the participation of other regulators of rRNA biosynthesis not examined in the present study.

### Altered expression of ribosomal protein mRNAs in PD is region- and stage-dependent

Ribosomes (80S) are cytoplasmic structures measuring 25–30 nm composed of 65 % RNAs and 35 % ribosomal proteins that form a smaller subunit (40S) which binds to mRNA and a larger subunit (60S) which binds to tRNAs and amino acids. In eukaryotes, the smaller subunit is made of 18S RNA and 33 proteins whereas the larger subunit is formed by 5S RNA, 5.8S RNA, 28S RNA, and 46 ribosomal proteins [55, 75–84]. Ribosomal proteins also participate in protein synthesis initiation and elongation, and they can regulate their own synthesis at the translational level [85–90].

The present results show decreased gene expression of 12 of 16 examined ribosomal protein mRNAs in the substantia nigra at stages 3–4 (covering 6 RPL and 6 RPS), and 14 (covering 8 RPL and 6 RPS) of 16 at stages 5–6 of Braak. These changes are progression-dependent and may reflect in part progressive neuronal loss in the substantia nigra. However, altered ribosomal protein gene expression also occurs in cerebral cortex in PD with stage and region peculiarities. Altered mRNA expression of several ribosomal proteins in the cerebral cortex appears to be a plastic process depending on the cortical region and stage of the disease. Decreased expression of a few mRNAs at stages 3–4 followed by up-regulation of a few different ribosomal protein mRNAs at stages 5–6 in frontal cortex and precuneus suggests modifications in the structure and functional capacities of cortical ribosomes with disease progression. It is worth stressing that only 16 of 79 ribosomal protein mRNAs were selected for the present study, and although the number is representative of ribosomal protein mRNA modifications it does not cover the total number of ribosomal proteins and the possible modifications of additional mRNAs.

### Expression of initiation and elongation factors in substantia nigra and frontal cortex area 8 in PD

Translation initiation in the ribosome is geared by the interactions of 12 eukaryotic translation initiation factors (eIFs), most of them composed of several subunits. The 43S preinitiation complex is composed of the small 40S ribosomal subunit, the initiating methionyl-tRNA bound to eIF2-GTP, and eIF1, eIF1a, and eIF3. mRNA is added to the 43S preinitiation complex together with the poly(A) binding protein (PABP) and the eIF4f complex (a heterotrimeric complex composed of eIF4a, eIF4e, and eIF4g) bound to an AUG codon. eIF2B and eIF5 activate eIF2 and regulate eIF2-GDP recycling, respectively, whereas eIF5b and eIF6 participate in ribosomal subunit joining and binding [91–94]. Elongation occurs when elongating factor eEF1A is activated following GTP-binding and forms a complex with aminoacyl-tRNA which recognizes the specific sequence in mRNA at the ribosome. Once the interaction of the codon in mRNA with the anti-codon in tRNA is decoded, eEF1A-GDP is hydrolysed, released from the ribosome, and recycled into its active form by eEF1B. eEF2 assists in the precise codon location at the ribosome [95–104]. Synthesis terminates in the presence of a stop codon in the mRNA sequence which is recognized by a releasing factor that sets the polypeptide chain free [105, 106].

In the substantia nigra, eIF3 and eEF2 expression levels were increased more markedly at stages 5–6 in PD, suggesting activation of peptide synthesis. It can be suggested that activation of peptide synthesis is related to compensatory mechanisms in preserved dopaminergic neurons in the face of the altered expression of genes involved in ribosomal proteins and, consequently, in the assembly of the functional ribosome. Alternatively, since western blots cannot discriminate between neurons and glial cells, increased eIF3 and eEF2 expression levels might be related to increased protein synthesis in reactive astrocytes.

In cerebral cortex area 8, among the five eIFs and subunits examined, only eIF3 was significantly decreased at stages 3–4 and 5–6, suggesting that recruitment of mRNA to the 40S subunit is hampered as a result of lower eIF3 expression. Regarding elongation factors, significantly reduced expression of eEF1A and eEF2 with disease progression lends strong support to the hypothesis of altered polypeptide synthesis in frontal cortex in PD.

### Reticulum stress responses in the substantia nigra and frontal cortex in PD with disease progression

The unfolded protein response (UPR) designates the cellular response to the accumulation of abnormal proteins in the endoplasmic reticulum. The reaction can also be elicited by other factors such as hypoglycemia, hypoxia, acidosis, calcium, redox reactions, and a variety of natural compounds and drugs [18]. Control of protein folding at the endoplasmic reticulum (ER) is modulated by the chaperone glucose-regulated protein 78 (GRP78, also named immunoglobulin binding protein BIP), a member of the HSP70 family which, in non-stressed cells, binds to three ER transmembrane proteins: PKR-like ER kinase (PERK), inositol requiring kinase 1 (IRE1), and transcription factor 6 (ATF6) [107–110]. Glucose-regulated protein 94 is the HSP90-like protein in the lumen of the endoplasmic reticulum and therefore it chaperones secreted and membrane proteins [111, 112]. Accumulation of misfolded proteins in the endoplasmic reticulum activates PERK [113] and phosphorylates the α-subunit of eukaryotic initiation factor 2 (eIF2-α) at serine 51, resulting in decreased protein synthesis [114]. In addition, eIF2α phosphorylation sets off activating transcription factor 4 (ATF4), promoting DNA transcription of specific genes [115]. ER responses also involve the activation of inositol-requiring kinase 1 (IRE1) by dimerization and phosphorylation which activates the transcription factor Xbox binding protein (XBP1), which in turns activates transcription of stress genes in DNA [69, 116, 117]. Upon ER stress, full activating transcription factor 6 (ATF6) of 90 kDa moves to the Golgi complex where it is cleaved to form the active transcription factor of 50 kDa (ATF6-50 kDa: ATF6f), which translocates to the nucleus and activates transcription of stress genes [118]. Therefore, activation of ATF4, IRE1, and ATF6f increases the production of GRP78, GRP94, PERK, IRE1, XBP1, and ATF6, and stimulates the ER-associated degradation (ERAD) pathway [119, 120], contributing to restoring homeostasis. However, once passed certain thresholds, ER stress can trigger NF-κB activation and caspase-mediated apoptosis [113, 121–123].

ER stress has been implicated in the pathogenesis of neurodegenerative diseases including PD [6, 124, 125]. Markers of the unfolded protein response (phosphorylated PERK and phosphorylated eIF2α) have been identified in dopaminergic neurons of the substantia nigra containing α-synuclein inclusions at relatively early stages of PD [126, 127]. ATF4 and total ATF6 expression levels are elevated in the substantia nigra at advanced stages of the disease, suggesting activation of the UPS response. It is worth noting that ATF4 levels have been reported to be increased in neuromelanin-containing neurons in the substantia nigra in PD, and elevated levels of ATF4 are protective of neurons subjected to noxious stimuli [128].

### α-synuclein oligomeric species

α-synuclein was first described in the nucleus and presynaptic nerve terminals from *Torpedo* [129]. The localization and function of this protein in the nucleus has been a primary focus of study partly due to the overwhelming information about the accumulation of abnormal α-synuclein in the cytoplasm of neurons in PD and in neurons and oligodendroglia in multiple system atrophy. However, α-synuclein is identified in the nucleus in different settings using different methods [130–136], and it is especially abundant during development modulating neurogenesis [137–139]. Nuclear α-synuclein levels are increased accompanying oxidative stress *in vitro* and *in vivo* [140, 141], and nuclear α-synuclein seems to facilitate, in turn, oxidative stress [142]. The mechanism of effects of nuclear α-synuclein is poorly understood but α-synuclein binds to histones and inhibits histone acetylation [130, 143]. Moreover, α-synuclein, under conditions of oxidative stress, binds to the promoter of the mitochondrial transcription factor PGC1-α and reduces transcription of mitochondrial genes [144].

α-synuclein is primarily a disordered monomer that binds to and transiently stabilizes different substrates such as lipids and membrane vesicles playing variegated physiological functions [145–147]. However, soluble β-rich oligomers are experimentally promoted *in vitro* by several physical and chemical agents, and they are also naturally produced in disease states influenced by fatty acids, α-synuclein mutations, oxidative stress, phosphorylation, nitration, ubiquitination, and truncation, where they come to be toxic for nerve cells [144, 148–154]. Increased expression levels of α-synuclein oligomers have been found in the brain in Lewy body diseases and related transgenic models [14, 155–158]. Interestingly, the band pattern of α-synuclein oligomers analysed here differs in the substantia nigra and frontal cortex area 8, suggesting regional differences in the composition of oligomeric species in the substantia nigra and cerebral cortex in PD. Importantly, the intensity of oligomeric species in western blots is greater than what is expected following examination of paraformaldehyde-fixed paraffin sections of the frontal cortex in which only a few Lewy bodies and neurites are detected with immunohistochemistry using the same antibody. Therefore, it may be inferred that most α-synuclein oligomers are not identified in paraffin sections processed for immunohistochemistry; only those linked to fibrillary deposits in Lewy bodies and neurites remain. Observations in the putamen are particularly interesting as no oligomeric α-synuclein species are identified in the same PD cases used in substantia nigra and frontal cortex. This means important regional differences in α-synuclein oligomers in PD represent, on the one hand, regional differences in α-synuclein metabolism, and, on the other, these differences may reflect specific regional vulnerability in PD.

A recent study has shown that α-synuclein proximity ligation assay (AS-PLA) permits the visualization of undetected diffuse α-synuclein oligomeric pathology in PD brains [159]. α-synuclein oligomers are detected in the cytoplasm of neurons with α-synuclein inclusions, as revealed with current immunohistochemistry, but also widespread in nerve terminals (consistent with synaptic localization) and in the cytoplasm of vulnerable neurons with no apparent α-synuclein pathology as detected by current immunohistochemical methods. No nuclear α-synuclein oligomers were reported in that study but the accompanying figures showed small immunoreactive dots in certain nuclei. The present observations show unequivocal presence of α-synuclein oligomers in FACS-isolated neuronal nuclei in PD. Interestingly, a weak band of α-synuclein is also detected in NeuN- samples (corresponding to non-neuronal nuclei) in PD. This raises the possibility that α-synuclein is abnormally present in the nuclei of glial cells in PD. In this line, α-synuclein deposition has been reported in protoplasmic astrocytes in PD [160, 161]. It is worth stressing that weak bands of α-synuclein oligomers in isolated neuronal nuclei from certain MA are only visualized after long-term exposure. AS-PLA has also allowed the visualization of α-synuclein oligomers in neuronal nuclei in control cases (see Fig. 12, ref 131) although this fact was not mentioned in the original paper.

### Conclusions

Our previous studies, among others, have shown that in spite of the absence or the relatively small numbers of Lewy bodies and neurites in the cerebral cortex in PD until advanced stages of the disease, there is a plethora of molecular alterations including altered synaptic modulation and transmission, mitochondrial dysfunction, oxidative stress damage, reduced energy metabolism, altered purine metabolism, increased inflammatory responses, and abnormal expression of receptors whose function is still poorly understood [13–15, 162, 163]. All these alterations converge in the most vulnerable regions of the cerebral cortex and extend to other regions with disease progression [126, 164–166]. To the list of apparently unrelated deleterious events, we may add altered machinery of protein synthesis targeted not only in the substantia nigra but also in the cerebral cortex in PD at middle and advanced stages of the disease. Frontal cortex area 8 is more affected than the angular gyrus and precuneus. α-synuclein oligomeric species seem to have direct and indirect deleterious effects on mitochondria [152, 167–169], proteasome [170], endoplasmic reticulum [6], and synapses [171–173], among other subcellular structures [153, 174]. Importantly, altered protein machinery in PD relates to the presence of α-synuclein oligomeric species in total homogenates. Substantia nigra and frontal cortex are enriched, albeit with different band patterns, in α-synuclein oligomeric species, whereas α-synuclein oligomers are not detected in the putamen. Unfortunately, rapid blocking of protein synthesis following hypoxia and other insults aimed at not producing abnormal proteins under suboptimal conditions such as those inherent to the process of dying precludes direct and accurate study of protein synthesis in human post-mortem brains (unpublished observations). Therefore, a direct observation of impaired protein synthesis in PD post-mortem brains is technically not possible.

## Additional files

Additional file 1: Table S1. Summary of the main individual characteristics of the cases used in this study. M: male; F: female; P-M: post-mortem delay (hours, minutes); PD Braak: Parkinson’s disease-related pathology stages 1–6 of Braak; 0: no neurological or neuropathological anomalies; FC: frontal cortex area 8; SN: substantia nigra; AG: angular gyrus; PC: precuneus; PUT: putamen; RIN: RNA integrity number; WB: western blot; IHC: immunohistochemistry; OLI: α-synuclein oligomeric species. (DOC 331 kb) Additional file 2: Table S2. TaqMan probes used for the study of mRNA expression of nucleolar proteins, ribosomal RNAs, and ribosomal proteins including the probes for normalization (GUS-β and XPNPEP1). (DOC 42 kb) Additional file 3: Table S3. Summary of the antibodies used for western-blotting (wb) and immunohistochemistry (ihq) or immunofluorescence (if); rb: rabbit polyclonal: m: mouse monoclonal; ip: immunoprecipitation for FACS studies. (DOC 54 kb) Additional file 4: Table S4. Expression levels of mRNAs encoding nucleolar proteins 18S rRNA and 28S rRNA, and mRNAs encoding ribosomal proteins in the substantia nigra. MA: middle-aged individuals with no PD pathology, 1–6 stages of PD. (DOC 50 kb) Additional file 5: Table S5. Expression levels of mRNAs encoding nucleolar proteins 18S rRNA and 28S rRNA, and mRNAs encoding ribosomal proteins in frontal cortex area 8. MA: middle-aged individuals with no PD pathology, 1–6 stages of PD. (DOC 58 kb) Additional file 6: Table S6. Expression levels of mRNAs encoding nucleolar proteins 18S rRNA and 28S rRNA, and mRNAs encoding ribosomal proteins in the angular gyrus. MA: middle-aged individuals with no PD pathology, 1–6 stages of PD (DOC 58 kb) Additional file 7: Table S7. Expression levels of mRNAs encoding nucleolar proteins 18S rRNA and 28S rRNA, and mRNAs encoding ribosomal proteins in the precuneus. MA: middle-aged individuals with no PD pathology, 1–6 stages of PD (DOC 58 kb) Additional file 8: Table S8. Expression levels of mRNAs encoding nucleolar proteins 18S rRNA and 28S rRNA, and mRNAs encoding ribosomal proteins in the putamen. MA: middle-aged individuals with no PD pathology, 1–6 stages of PD (DOC 48 kb) Additional file 9: Table S9. Mean ratio of the number of nucleolar staining and the total number of neurons (ratio SD) visualized with haematoxylin and eosin and immunohistochemistry to NPM1 and NPM3 in the substantia nigra at stages 1, 3, 4, and 5 of PD. Percentage (%) of nucleolus staining and total neurons. No significant differences are seen regarding the ratios of NPM3 nucleolar staining along disease progression. However, NPM1 immunohistochemistry reveals a significant decrease between PD1 and PD5 (P ≤ 0.05 One-way Anova) (DOC 28 kb) Additional file 10: Figure S1. Western blotting of isolated nuclei obtained by FACS of frontal cortex area 8 of middle-aged (MA) and Parkinson’s disease (PD) stage 5–6 cases subjected to over-exposure (10 min). Several bands of α-synuclein oligomeric species are seen in PD as in Figure 9B (exposure 1 min). In addition, weak bands of α-synuclein oligomers are observed in isolated neuronal nuclei (NeuN+) in MA. (TIF 426 kb)

## Abbreviations

ATF4: activating transcription factor 4
ATF6: activating transcription factor 6
eIF1: eukaryotic translation initiation factor 1
eIF2-α: eukaryotic translation initiation factor 2
eIF3: eukaryotic translation initiation factor 3
eIF3η: eukaryotic translation initiation factor 3 η
eIF5: eukaryotic translation initiation factor 5
eEF1A: eukaryotic elongation factor 1A
eEF2: eukaryotic elongation factor 2
GRP94: glucose-regulated protein 94
GRP78: glucose-regulated protein 78
GUS-B: β-glucuronidase
IRE-1: inositol requiring kinase 1
NCL: nucleolin
NPM1: nucleophosmin 1 (nucleolar phosphoprotein B23)
NPM3: nucleophosmin/nucleoplasmin 3
P-IRE-1: phosphorylated inositol requiring kinase 1
P-eIF2-α: phosphorylated eukaryotic translation initiation factor 2
RPL5: ribosomal protein L5
RPL7: ribosomal protein L7
RPL21: ribosomal protein L21
RPL22: ribosomal protein L22
RPL23A: ribosomal protein L23A
RPL26: ribosomal protein L26
RPL27: ribosomal protein L27
RPL30: ribosomal protein L30
RPL31: ribosomal protein L31
RPS3A: ribosomal protein S3A
RPS5: ribosomal protein S5
RPS6: ribosomal protein S6
RPS10: ribosomal protein S10
RPS13: ribosomal protein S13
RPS16: ribosomal protein S16
RPS17: ribosomal protein S17
rRNA 28S: ribosomal RNA subunit 28
rRNA18S: ribosomal RNA subunit 18
UBTF: UBF, upstream binding transcription factor, RNA polymerase I
XBP1: Xbox binding protein 1
XPNPEP1: X-prolylaminopeptidase P1
