# Supplementary material for: Altered machinery of protein synthesis is region- and stage-dependent and is associated with α-synuclein oligomers in Parkinson’s disease
Source: Acta Neuropathol Commun. 2015 Dec 1;3:76. doi: 10.1186/s40478-015-0257-4 (PMC4666041; doi:10.1186/s40478-015-0257-4)
Supplement: Additional file 1: Table S1. — Summary of the main individual characteristics of the cases used in this study. M: male; F: female; P-M: post-mortem delay (hours, minutes); PD Braak: Parkinson’s disease-related pathology stages 1–6 of Braak; 0: no neurological or neuropathological anomalies; FC: frontal cortex area 8; SN: substantia nigra; AG: angular gyrus; PC: precuneus; PUT: putamen; RIN: RNA integrity number; WB: western blot; IHC: immunohistochemistry; OLI: α-synuclein oligomeric species. (DOC 331 kb) [file 40478_2015_257_MOESM1_ESM.doc]

**Supplementary Table I**: Summary of the main individual characteristics of the cases used in this study. M: male; F: female; P-M: post-mortem delay (hours, minutes); PD Braak: Parkinson’s disease-related pathology stages 1-6 of Braak; 0: no neurological or neuropathological anomalies; FC: frontal cortex area 8; SN: substantia nigra; AG: angular gyrus; PC: precuneus; PUT: putamen; RIN: RNA integrity number; WB: western blot; IHC: immunohistochemistry; OLI: α-synuclein oligomeric species.

| **Nº CASE** | **GENDER** | **AGE** | **P-M** | **PD BRAAK** | **SN** | **RIN** | **FC** | **RIN** | **AG** | **RIN** | **PC** | **RIN** | **PUT** | **RIN** | **WB FC** | **WB SN** | **IHC** | **OLI SN** | **OLI FC** |
| --- | --- | --- | --- | --- | --- | --- | --- | --- | --- | --- | --- | --- | --- | --- | --- | --- | --- | --- | --- |
| 1 | M | 64 | 8h30m | 0 |  |  | X | 7.7 | X | 7.7 |  |  |  |  | X |  |  |  |  |
| 2 | M | 67 | 14h40m | 0 | X | 6.5 |  |  |  |  |  |  |  |  |  |  |  | X |  |
| 3 | M | 56 | 5h | 0 |  |  | X | 7.8 | X | 6.8 |  |  |  |  | X |  |  |  |  |
| 4 | M | 67 | 5h | 0 |  |  | X | 7.4 | X | 6.7 | X | 7.3 |  |  | X |  |  |  |  |
| 5 | M | 62 | 3h | 0 |  |  | X | 8 | X | 6.8 | X | 7.8 |  |  | X |  |  |  | X |
| 6 | M | 52 | 4h40m | 0 |  |  | X | 8.1 | X | 6.7 | X | 6.4 |  |  | X |  |  |  |  |
| 7 | M | 30 | 4h10m | 0 |  |  | X | 8.4 |  |  | X | 8 |  |  | X |  |  |  |  |
| 8 | M | 53 | 3h | 0 |  |  | X | 8.8 | X | 6.6 |  |  |  |  | X |  |  |  | X |
| 9 | F | 49 | 7h | 0 |  |  | X | 8.2 |  |  |  |  |  |  | X |  |  |  |  |
| 10 | F | 75 | 3h | 0 |  |  | X | 7.7 | X | 8.7 |  |  |  |  | X |  |  |  | X |
| 11 | F | 46 | 9h35m | 0 |  |  | X | 6.8 | X | 6.8 |  |  |  |  | X |  |  |  |  |
| 12 | F | 86 | 4h15m | 0 |  |  | X | 8.1 |  |  | X | 8.2 | X | 6.9 | X |  |  |  |  |
| 13 | F | 79 | 3h35m | 0 |  |  | X | 7.7 |  |  |  |  | X | 7.8 | X |  |  |  |  |
| 14 | F | 79 | 6h25m | 0 | X | 6.2 | X | 7.8 |  |  | X | 6.7 |  |  | X |  |  |  |  |
| 15 | F | 77 | 3h15m | 0 |  |  | X | 7 |  |  |  |  |  |  | X |  |  |  |  |
| 16 | F | 76 | 5h45m | 0 |  |  | X | 8.3 |  |  |  |  |  |  |  |  |  |  |  |
| 17 | F | 71 | 8h30m | 0 |  |  | X | 7.4 |  |  | X | 6.7 |  |  |  |  |  |  |  |
| 18 | M | 59 | 4h15m | 0 | X | 6.8 |  |  |  |  | X | 8.2 | X | 6.7 |  |  |  |  |  |
| 19 | M | 70 | 2h | 0 | X | 6.5 |  |  |  |  |  |  |  |  |  |  |  |  |  |
| 20 | M | 61 | 4h30h | 0 | X | 7.1 |  |  |  |  | X | 6.7 | X | 6.5 |  |  |  |  |  |
| 21 | M | 63 | 8h | 0 | X | 6.4 |  |  |  |  |  |  | X | 7.2 |  |  |  |  |  |
| 22 | M | 30 | 4h10m | 0 | X | 7.4 |  |  |  |  |  |  |  |  |  |  |  |  |  |
| 23 | M | 57 | 4h30m | 0 | X | 6.6 |  |  |  |  |  |  | X | 6.9 |  |  |  |  |  |
| 24 | M | 60 | 4h15m | 0 | X | 6.6 |  |  |  |  |  |  | X | 7.3 |  |  |  |  |  |
| 25 | F | 68 | 4h30m | 0 | X | 6 |  |  |  |  |  |  |  |  |  |  |  |  |  |
| 26 | F | 64 | 2h15m | 0 | X | 7.3 |  |  |  |  |  |  | X | 8.7 |  |  |  |  |  |
| 27 | F | 46 | 9h35m | 0 | X | 7.2 |  |  |  |  |  |  |  |  |  |  |  |  |  |
| 28 | M | 77 | 6h55m | 0 |  |  |  |  |  |  | X | 7.9 | X | 6.5 |  |  |  |  |  |
| 29 | M | 78 | 12h | 0 |  |  |  |  |  |  |  |  | X | 6.9 |  |  |  |  |  |
| 30 | F | 72 | 4h | 0 |  |  |  |  |  |  |  |  | X | 6.7 |  |  |  |  |  |
| 31 | M | 75 | 5h15m | 0 |  |  |  |  |  |  |  |  | X | 7 |  |  |  |  |  |
| 32 | M | 76 | 4h15m | 0 |  |  |  |  |  |  |  |  | X | 6.8 |  |  |  |  |  |
| 33 | M | 71 | 5h15m | 0 |  |  |  |  |  |  |  |  | X | 7.9 |  |  |  |  |  |
| 34 | M | 76 | 4h15m | 0 |  |  |  |  |  |  |  |  | X | 6.6 |  |  |  |  |  |
| 35 | M | 56 | 5h | 0 |  |  |  |  | X | 6.9 |  |  |  |  |  |  |  |  |  |
| 36 | F | 78 | 12h | 0 |  |  |  |  | X | 6.5 |  |  |  |  |  |  |  |  |  |
| 37 | M | 67 | 11h45m | 0 |  |  |  |  |  |  | X | 7.8 |  |  |  |  |  |  |  |
| 38 | F | 79 | 10h | 0 |  |  |  |  |  |  |  |  |  |  |  | X |  |  |  |
| 39 | F | 78 | 3h15m | 0 |  |  |  |  |  |  |  |  |  |  |  | X |  |  |  |
| 40 | M | 75 | 3h25m | 0 |  |  |  |  |  |  |  |  |  |  |  | X |  |  |  |
| 41 | M | 76 | 6h | 0 |  |  |  |  |  |  |  |  |  |  |  | X |  |  |  |
| 42 | M | 75 | 7h55m | 0 |  |  |  |  |  |  |  |  |  |  |  | X |  |  |  |
| 43 | M | 81 | 5h5m | 0 |  |  |  |  |  |  |  |  |  |  |  | X |  |  |  |
| 44 | F | 73 | 15h45m | 0 |  |  |  |  |  |  |  |  |  |  |  | X |  | X |  |
| 45 | M | 74 | 10h50m | PD 1 | X | 6.4 |  |  |  |  |  |  |  |  |  |  |  |  |  |
| 46 | M | 80 | 6h | PD 1 | X | 6.5 |  |  |  |  |  |  |  |  |  |  |  |  |  |
| 47 | M | 72 | 8h55m | PD 1 |  |  |  |  |  |  |  |  |  |  |  |  | X |  |  |
| 48 | F | 75 | 23h | PD 1 |  |  |  |  |  |  |  |  |  |  |  |  | X |  |  |
| 49 | M | 83 | 3h30m | PD 2 | X | 6.6 |  |  |  |  |  |  |  |  |  |  |  |  |  |
| 50 | F | 97 | 3h40m | PD 2 | X | 6.4 |  |  |  |  |  |  |  |  |  |  |  |  |  |
| 51 | M | 57 | 11h | PD 3 |  |  | X | 8.7 | X | 8.3 |  |  |  |  | X |  |  |  |  |
| 52 | F | 54 | 11h10m | PD 3 |  |  | X | 8.2 |  |  |  |  |  |  | X |  |  |  |  |
| 53 | M | 81 | 4h55m | PD 3 | X | 6.9 |  |  |  |  |  |  | X | 6.7 |  |  | X |  |  |
| 54 | M | 71 | 18h45m | PD 3 |  |  |  |  |  |  |  |  |  |  |  |  | X |  |  |
| 55 | M | 73 | 4h15m | PD 3 |  |  |  |  |  |  |  |  |  |  |  |  | X |  |  |
| 56 | F | 70 | 10h50m | PD 3 |  |  |  |  | X | 8.7 |  |  |  |  |  |  |  |  |  |
| 57 | F | 89 | 18h10m | PD 3 |  |  |  |  |  |  |  |  |  |  |  | X |  |  |  |
| 58 | M | 69 | 5h55m | PD 4 |  |  |  |  |  |  |  |  |  |  |  |  | X |  |  |
| 59 | M | 66 | 5h | PD 4 |  |  | X | 7.7 | X | 7.3 |  |  |  |  | X |  |  |  |  |
| 60 | M | 57 | 19h | PD 4 |  |  | X | 7.6 | X | 7 |  |  |  |  | X |  |  |  |  |
| 61 | M | 76 | 4h30m | PD 4 |  |  | X | 7.7 | X | 7.9 | X | 7.7 |  |  | X |  |  |  |  |
| 62 | M | 68 | 4h45m | PD 4 |  |  | X | 7.3 | X | 8.5 | X | 8.3 |  |  | X |  |  |  |  |
| 63 | M | 79 | 9h15m | PD 4 |  |  | X | 7.2 |  |  |  |  |  |  | X |  |  |  |  |
| 64 | M | 69 | 5h55m | PD 4 |  |  | X | 6.3 | X | 7.6 |  |  |  |  | X |  |  |  |  |
| 65 | F | 84 | 4h30m | PD 4 | X | 6.8 | X | 7.8 |  |  |  |  |  |  |  |  |  |  |  |
| 66 | M | 68 | 9h20m | PD 4 |  |  | X | 6.9 |  |  |  |  |  |  |  |  |  |  |  |
| 67 | M | 77 | 12h | PD 4 |  |  | X | 6.9 |  |  |  |  |  |  |  |  |  |  |  |
| 68 | M | 68 | 9h20m | PD 4 | X | 8.2 |  |  |  |  |  |  |  |  |  |  |  |  |  |
| 69 | M | 80 | 7h30m | PD 4 | X | 7.1 |  |  |  |  |  |  |  |  |  |  |  |  | X |
| 70 | M | 85 | 11h45m | PD 4 | X | 7.7 |  |  |  |  |  |  |  |  |  |  |  |  |  |
| 71 | F | 70 | 4h40m | PD 4 |  |  |  |  |  |  | X | 8 | X | 6.5 |  | X |  |  |  |
| 72 | M | 84 | 9h | PD 4 |  |  |  |  |  |  |  |  | X | 6.5 |  | X |  |  |  |
| 73 | M | 74 | 6h45m | PD 4 |  |  |  |  |  |  | X | 6.9 | X | 7.3 |  |  | X |  |  |
| 74 | F | 88 | 11h50m | PD 4 |  |  |  |  |  |  | X | 6.8 | X | 7.2 |  |  |  |  |  |
| 75 | M | 85 | 11h45m | PD 4 |  |  |  |  |  |  | X | 6.2 | X | 6.9 |  |  |  |  |  |
| 76 | M | 50 | 9h15m | PD 4 |  |  |  |  |  |  |  |  | X | 6.3 |  |  |  |  |  |
| 77 | M | 69 | 15h05m | PD 4 |  |  |  |  | X | 8.1 |  |  |  |  |  |  |  |  |  |
| 78 | M | 85 | 3h15m | PD 4 |  |  |  |  |  |  | X | 6.5 |  |  |  |  |  |  |  |
| 79 | F | 70 | 5h15m | PD 4 |  |  |  |  |  |  | X | 6 |  |  |  |  |  |  |  |
| 80 | M | 83 | 4h | PD 4 |  |  |  |  |  |  | X | 6.6 |  |  |  |  |  |  |  |
| 81 | M | 69 | 15h5m | PD 4 |  |  |  |  |  |  | X | 6 |  |  |  |  | X |  |  |
| 82 | F | 84 | 4h30m | PD 4 | X | 7.9 |  |  |  |  |  |  |  |  |  |  |  |  |  |
| 83 | M | 77 | 6h15m | PD 4 | X | 7.7 |  |  |  |  |  |  |  |  |  |  |  |  |  |
| 84 | F | 81 | 6h30m | PD 4 | X | 6.7 |  |  |  |  |  |  |  |  |  |  |  |  |  |
| 85 | F | 69 | 5h10m | PD 4 | X | 6.6 |  |  |  |  |  |  |  |  |  |  |  |  |  |
| 86 | M | 71 | 5h | PD 4 | X | 6.8 |  |  |  |  |  |  |  |  |  |  |  |  |  |
| 87 | M | 81 | 7h20m | PD 4 | X | 7 |  |  |  |  |  |  |  |  |  |  |  |  |  |
| 88 | F | 83 | 4h | PD 4 | X | 7.7 |  |  |  |  |  |  |  |  |  |  |  |  |  |
| 89 | M | 76 | 4h25m | PD 4 |  |  |  |  |  |  |  |  |  |  |  | X |  |  |  |
| 90 | M | 65 | 6h30m | PD 4 |  |  |  |  |  |  |  |  |  |  |  | X |  |  |  |
| 91 | F | 70 | 4h30m | PD 5 |  |  | X | 8.5 |  |  |  |  |  |  | X |  |  |  |  |
| 92 | F | 77 | 3h30m | PD 5 |  |  | X | 6.8 |  |  |  |  |  |  | X |  |  |  |  |
| 93 | F | 81 | 6h30m | PD 5 |  |  | X | 8.3 |  |  |  |  |  |  | X |  |  |  |  |
| 94 | F | 69 | 4h30m | PD 5 |  |  | X | 8 |  |  |  |  |  |  | X |  |  |  |  |
| 95 | F | 79 | 3h30m | PD 5 |  |  | X | 8 |  |  |  |  |  |  | X |  |  |  |  |
| 96 | M | 78 | 13h30m | PD 5 | X | 6 | X | 6.7 |  |  |  |  |  |  |  |  |  |  |  |
| 97 | M | 83 | 14h | PD 5 | X | 7.6 | X | 7.7 |  |  |  |  |  |  |  |  |  |  |  |
| 98 | F | 77 | 7h30m | PD 5 | X | 6 | X | 7.1 |  |  |  |  |  |  |  |  |  |  |  |
| 99 | M | 76 | 12h | PD 5 | X | 6.7 |  |  |  |  |  |  |  |  |  |  |  |  | X |
| 100 | M | 84 | 16h30m | PD 5 | X | 6.3 |  |  |  |  | X | 6.1 |  |  |  | X | X |  |  |
| 101 | M | 79 | 4h30m | PD 5 |  |  |  |  |  |  | X | 6.5 | X | 6.5 |  |  | X |  |  |
| 102 | M | 77 | 7h30m | PD 5 |  |  |  |  |  |  | X | 6.9 | X | 6.6 |  |  | X |  |  |
| 103 | F | 78 | 4h30m | PD 5 |  |  |  |  | X | 6.6 |  |  |  |  |  |  |  |  |  |
| 104 | F | 79 | 1h30m | PD 5 |  |  |  |  | X | 7.2 |  |  |  |  |  |  |  |  |  |
| 105 | M | 76 | 4h | PD 5 |  |  |  |  | X | 6.6 |  |  |  |  |  |  |  |  |  |
| 106 | M | 84 | 4h | PD 5 |  |  |  |  | X | 7.7 |  |  |  |  |  |  |  |  |  |
| 107 | F | 85 | 6h15m | PD 5 |  |  |  |  |  |  | X | 6.4 |  |  |  |  |  |  |  |
| 108 | F | 87 | 6h | PD 5 | X | 7 |  |  |  |  |  |  |  |  |  |  |  |  |  |
| 109 | M | 81 | 7h | PD 5 | X | 7.6 |  |  |  |  |  |  |  |  |  |  |  |  |  |
| 110 | F | 77 | 7h30m | PD 5 | X | 7.5 |  |  |  |  |  |  |  |  |  |  |  |  |  |
| 111 | F | 88 | 7h | PD 5 | X | 7.4 |  |  |  |  |  |  |  |  |  |  |  |  |  |
| 112 | M | 81 | 5h | PD 5 | X | 7.6 |  |  |  |  |  |  |  |  |  |  |  |  |  |
| 113 | M | 74 | 8h | PD 5 | X | 7.5 |  |  |  |  |  |  |  |  |  |  |  |  |  |
| 114 | M | 78 | 5h15m | PD 5 | X | 6.7 |  |  |  |  |  |  |  |  |  |  |  |  |  |
| 115 | M | 60 | 8h | PD 5 |  |  |  |  |  |  |  |  |  |  |  | X |  |  |  |
| 116 | M | 50 | 16h30m | PD 5 |  |  |  |  |  |  |  |  |  |  |  |  |  |  | X |
| 117 | M | 80 | 7h30m | PD 6 | X | 7.9 | X | 7.5 |  |  |  |  |  |  |  |  |  |  |  |
| 118 | M | 80 | 7h30m | PD 6 | X | 6.9 |  |  |  |  |  |  |  |  |  |  |  |  |  |
| 119 | M | 61 | 4h30m | PD 6 |  |  |  |  |  |  |  |  |  |  |  |  |  | X |  |
| 120 | M | 75 | 3h40m | PD 6 |  |  |  |  |  |  |  |  |  |  |  |  |  | X |  |
| 121 | F | 89 | 5h10m | PD 6 |  |  |  |  |  |  |  |  |  |  |  |  |  | X |  |
| 122 | M | 80 | 8h | PD 6 |  |  |  |  |  |  |  |  |  |  |  |  |  | X |  |
