# Supplementary material for: Altered machinery of protein synthesis is region- and stage-dependent and is associated with α-synuclein oligomers in Parkinson’s disease
Source: Acta Neuropathol Commun. 2015 Dec 1;3:76. doi: 10.1186/s40478-015-0257-4 (PMC4666041; doi:10.1186/s40478-015-0257-4)
Supplement: Additional file 2: Table S2. — TaqMan probes used for the study of mRNA expression of nucleolar proteins, ribosomal RNAs, and ribosomal proteins including the probes for normalization (GUS-β and XPNPEP1). (DOC 42 kb) [file 40478_2015_257_MOESM2_ESM.doc]

**Supplementary Table II**: TaqMan probes used for the study of mRNA expression of nucleolar proteins, ribosomal RNAs, and ribosomal proteins including the probes for normalization (GUS-β and XPNPEP1).

| **Gene** | **Full Name** | **Reference** |
| --- | --- | --- |
| *GUS-B* | β-glucuronidase | Hs_00939627_m1 |
| *XPNPEP1* | X-prolylaminopeptidase (aminopeptidase P) 1 | Hs_00958026_m1 |
| *NCL* | nucleolin | Hs_01066668_m1 |
| *NPM1* | nucleophosmin (nucleolar phospho-protein B23, numatrin) | Hs_02339479_m1 |
| *NPM3* | nucleophosmin / nucleoplasmin 3 | Hs_00199625_m1 |
| *rRNA 28S* | RNA, 28S ribosomal 5 | Hs_03654441_s1 |
| *rRNA 18S* | eukaryotic 18S rRNA | Hs_99999901_s1 |
| *UBTF* | upstream binding transcription factor, RNA polymerase I | Hs_01115792_g1 |
| *RPL5* | ribosomal protein L5 | Hs_03044958_g1 |
| *RPL7* | ribosomal protein L7 | Hs_02596927_g1 |
| *RPL21* | ribosomal protein L21 | Hs_00823333_s1 |
| *RPL22* | ribosomal protein L22 | Hs_01865331_s1 |
| *RPL23A* | ribosomal protein L23A | Hs_01921329_g1 |
| *RPL26* | ribosomal protein L26 | Hs_00864008_m1 |
| *RPL27* | ribosomal protein L27 | Hs_03044961_g1 |
| *RPL30* | ribosomal protein L30 | Hs_00265497_m1 |
| *RPL31* | ribosomal protein L31 | Hs_0101549_g1 |
| *RPS3A* | ribosomal protein S3A | Hs_00832893_sH |
| *RPS5* | ribosomal protein S5 | Hs_00734849_g1 |
| *RPS6* | ribosomal protein S6 | Hs_04195024_g1 |
| *RPS10* | ribosomal protein S10 | Hs_01652370_gH |
| *RPS13* | ribosomal protein S13 | Hs_01011487_g1 |
| *RPS16* | ribosomal protein S16 | Hs_01598516_g1 |
| *RPS17* | ribosomal protein S17 | Hs_00734303_g1 |
