# Supplementary material for: Altered machinery of protein synthesis is region- and stage-dependent and is associated with α-synuclein oligomers in Parkinson’s disease
Source: Acta Neuropathol Commun. 2015 Dec 1;3:76. doi: 10.1186/s40478-015-0257-4 (PMC4666041; doi:10.1186/s40478-015-0257-4)
Supplement: Additional file 3: Table S3. — Summary of the antibodies used for western-blotting (wb) and immunohistochemistry (ihq) or immunofluorescence (if); rb: rabbit polyclonal: m: mouse monoclonal; ip: immunoprecipitation for FACS studies. (DOC 54 kb) [file 40478_2015_257_MOESM3_ESM.doc]

**Supplementary Table III:** Summary of the antibodies used for western-blotting (wb) and immunohistochemistry (ihq) or immunofluorescence (if); rb: rabbit polyclonal: m: mouse monoclonal; ip: immunoprecipitation for FACS studies.

| **antibody** | **reference** | **supplier** | **host** | **wb** | **ihq** |
| --- | --- | --- | --- | --- | --- |
| anti-nucleophosmin 1 (NPM1) | ab10530 | Abcam (Cambridge, UK) | m | - | 1/200 |
| anti-nucleophosmin/nucleoplasmin 3 (NPM3) | NBP1-90999 | Novus Biologicals (Cambridge, UK) | rb | - | 1/250 |
| anti-α-synuclein | Ab5038 | Chemicon (Darmstadt, Germany) | rb | - | 1/500 |
| anti-α-synuclein (NAC) | ANC0603 | Atgen (Gyeonggi-do, South Korea) | m | - | 1/250 |
| anti-α-synuclein oligomer-specific | AS132718 | Agrisera (Vännäs, Sweden) | m | 1/1,000 | - |
| anti-eukaryotic translation initiation factor 1 (EIF1) | NBP2-00702 | Novus Biologicals (Cambridge, UK) | m | 1/ 500 | - |
| anti-eukaryotic translation initiation factor 2 (eIF2-α) | 5A5 | Thermo Scientific (Waltham, MA, USA) | m | 1/50 | - |
| anti-phospho-eIF2-alpha pSer51 (p-EIF2-α) | S.674.5 | Thermo Scientific (Waltham, MA, USA) | rb | 1/50 | - |
| anti-eukaryotic translation initiation factor 3 (EIF3) | ab69842 | Abcam (Cambridge, UK) | rb | 1/250 | - |
| anti-eukaryotic translation initiation factor 3η (eIF3η) | sc-28857 | Santa Cruz (Dallas, TX, USA) | rb | 1/200 | - |
| anti-eukaryotic translation initiation factor 5 (eIF5) | sc-282 | Santa Cruz (Dallas, TX, USA) | rb | 1/400 | - |
| anti-eukaryotic elongation factor 1A (eEF1A) | 2551 | Cell signaling (Danvers, MA, USA) | rb | 1/100 | - |
| anti-eukaryotic elongation factor 2 (eEF2) | 2332 | Cell signaling (Danvers, MA, USA) | m | 1/1,000 | - |
| anti-activating transcription factor 4 (ATF4) | ab1371 | Abcam (Cambridge, UK) | gt | 1/500 | 1/100 |
| anti-activating transcription factor 6 (ATF6) | ALX-804-381 | Enzo Life Sciences (Farmingdale, NY, USA) | m | 1/250 | 1/50 |
| anti-glucose-regulated protein, 78kDa (GRP78) | ab53068 | Abcam (Cambridge, UK) | rb | 1/800 | 1/100 |
| anti-glucose-regulated protein, 94kDa (GRP94) | ab3674 | Abcam (Cambridge, UK) | rb | 1/1,000 | 1/100 |
| anti-inositol requiring kinase 1 (IRE1) | 45-779 | Prosci incorporated (Poway, CA, USA) | gt | - | 1/100 |
| anti-phoshorlyated inositol requiring kinase 1 (P-IRE1) | ab48187 | Abcam (Cambridge, UK) | rb | - | 1/100 |
| anti-rck (p54) | PD009 | MBL (Woburn, UK) | rb | - | 1/100 |
| anti-X-box binding protein (XBP1) | ab37152 | Abcam (Cambridge, UK) | rb | 1/500 | 1/200 |
| anti-histone H3 nuclear loading (H3) | ab1791 | Abcam (Cambridge, UK) | rb | 1/20,000 | - |
| anti-neuronal nuclei (NeuN) | MAB377 | Chemicon (Darmstadt, Germany) | m | **ip** 1/1,000 | |
| anti-superoxide dismutase (SOD1) | NCL-SOD1 | Novocastra (Nussloch, Germany) | m | 1/1,000 | - |
| anti-β-actin | A5316 | Sigma-Aldrich (St Louis, MO, USA) | m | 1/30,000 | - |
