# Supplementary material for: Altered machinery of protein synthesis is region- and stage-dependent and is associated with α-synuclein oligomers in Parkinson’s disease
Source: Acta Neuropathol Commun. 2015 Dec 1;3:76. doi: 10.1186/s40478-015-0257-4 (PMC4666041; doi:10.1186/s40478-015-0257-4)
Supplement: Additional file 7: Table S7. — Expression levels of mRNAs encoding nucleolar proteins 18S rRNA and 28S rRNA, and mRNAs encoding ribosomal proteins in the precuneus. MA: middle-aged individuals with no PD pathology, 1–6 stages of PD (DOC 58 kb) [file 40478_2015_257_MOESM7_ESM.doc]

**Supplementary Table VII:** Expression levels of mRNAs encoding nucleolar proteins 18S rRNA and 28S rRNA, and mRNAs encoding ribosomal proteins in the precuneus. MA: middle-aged individuals with no PD pathology, 1-6 stages of PD
