# Supplementary material for: Altered machinery of protein synthesis is region- and stage-dependent and is associated with α-synuclein oligomers in Parkinson’s disease
Source: Acta Neuropathol Commun. 2015 Dec 1;3:76. doi: 10.1186/s40478-015-0257-4 (PMC4666041; doi:10.1186/s40478-015-0257-4)
Supplement: Additional file 9: Table S9. — Mean ratio of the number of nucleolar staining and the total number of neurons (ratio SD) visualized with haematoxylin and eosin and immunohistochemistry to NPM1 and NPM3 in the substantia nigra at stages 1, 3, 4, and 5 of PD. Percentage (%) of nucleolus staining and total neurons. No significant differences are seen regarding the ratios of NPM3 nucleolar staining along disease progression. However, NPM1 immunohistochemistry reveals a significant decrease between PD1 and PD5 (P ≤ 0.05 One-way Anova) (DOC 28 kb) [file 40478_2015_257_MOESM9_ESM.doc]

**Supplementary Table IX**: Mean ratio of the number of nucleolar staining and the total number of neurons (ratio SD) visualized with haematoxylin and eosin and immunohistochemistry to NPM1 and NPM3 in the substantia nigra at stages 1, 3, 4, and 5 of PD. Percentage (%) of nucleolus staining and total neurons. No significant differences are seen regarding the ratios of NPM3 nucleolar staining along disease progression. However, NPM1 immunohistochemistry reveals a significant decrease between PD1 and PD5 (P ≤ 0.05 One-way Anova)

|  | HE | NPM1 | | | | NPM3 | | | |
| --- | --- | --- | --- | --- | --- | --- | --- | --- | --- |
|  | PD1 | PD1 | PD3 | PD4 | PD5 | PD1 | PD3 | PD4 | PD5 |
| ratio±SD | 0.51±0.07 | 0.50± 0.09 | 0.44± 0.05 | 0.37± 0.17 | 0.34± 0.06 | 0.48± 0.03 | 0.52± 0.11 | 0.53±0.11 | 0.44 ±0.07 |
| % | 51% | 50% | 44% | 37% | 34% | 48% | 52% | 53% | 44% |
