# Supplementary figures and images for: Altered machinery of protein synthesis is region- and stage-dependent and is associated with α-synuclein oligomers in Parkinson’s disease
Source: Acta Neuropathol Commun. 2015 Dec 1;3:76. doi: 10.1186/s40478-015-0257-4 (PMC4666041; doi:10.1186/s40478-015-0257-4)

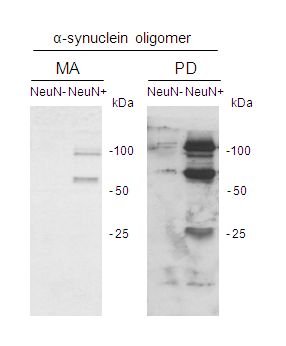

Supplement: Additional file 10: Figure S1. — Western blotting of isolated nuclei obtained by FACS of frontal cortex area 8 of middle-aged (MA) and Parkinson’s disease (PD) stage 5–6 cases subjected to over-exposure (10 min). Several bands of α-synuclein oligomeric species are seen in PD as in Figure 9B (exposure 1 min). In addition, weak bands of α-synuclein oligomers are observed in isolated neuronal nuclei (NeuN+) in MA. (TIF 426 kb) [file 40478_2015_257_MOESM10_ESM.tif]
